# Synthesis and Biological Activity Characterization of Novel 5-Oxopyrrolidine Derivatives with Promising Anticancer and Antimicrobial Activity

**DOI:** 10.3390/ph15080970

**Published:** 2022-08-06

**Authors:** Karolina Kairytė, Birutė Grybaitė, Rita Vaickelionienė, Birutė Sapijanskaitė-Banevič, Povilas Kavaliauskas, Vytautas Mickevičius

**Affiliations:** 1 Department of Organic Chemistry, Kaunas University of Technology, Radvilėnų Rd. 19, LT-50254 Kaunas, Lithuania; 2 Transplantation-Oncology Infectious Diseases Program, Division of Infectious Diseases, Department of Medicine, Weill Cornell Medicine of Cornell University, 527 East 68th Street, New York, NY 10065, USA; 3 Department of Microbiology and Immunology, University of Maryland Baltimore School of Medicine, 655 W. Baltimore Street, Baltimore, MD 21201, USA; 4 Biological Research Center, Veterinary Academy, Lithuanian University of Health Sciences, Tilžės St. 18, LT-47181 Kaunas, Lithuania; 5 Institute of Infectious Diseases and Pathogenic Microbiology, Birštono Str. 38A, LT-59116 Prienai, Lithuania

**Keywords:** azole, hydrazone, bishydrazone, pyrrolidinone, antimicrobial properties, biological activity, multidrug-resistant pathogens, *Staphylococcus aureus*, MRSA, A549, lung cancer

## Abstract

The 1-(4-acetamidophenyl)-5-oxopyrrolidine carboxylic acid was applied for synthesizing derivatives bearing azole, diazole, and hydrazone moieties in the molecule. Modification of an acetamide fragment to the free amino group afforded compounds with two functional groups, which enabled to provide a series of 4-substituted-1-(4-substituted phenyl)pyrrolidine-2-ones. The resulted compounds **2** and **4**–**22** were subjected to the in vitro anticancer and antimicrobial activity determination. The compounds **18**–**22** exerted the most potent anticancer activity against A549 cells. Furthermore, compound **21** bearing 5-nitrothiophene substituents demonstrated promising and selective antimicrobial activity against multidrug-resistant *Staphylococcus aureus* strains, including linezolid and tedizolid-resistant *S. aureus*. These results demonstrate that 5-oxopyrolidine derivatives are attractive scaffolds for the further development of anticancer and antimicrobial compounds targeting multidrug-resistant Gram-positive pathogens.

## 1. Introduction

Growing antimicrobial resistance among clinically significant pathogens is considered to be one of the major threats worldwide. Environmental exposure to numerous chemical agents is associated with a growing incidence of cancer. Therefore, it is important to develop novel bioactive molecules that could be further explored as anticancer or antimicrobial agents.

**Figure 1:**
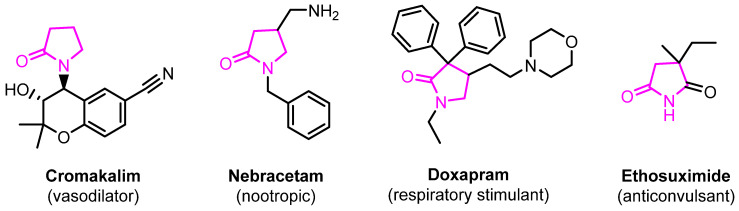
Pharmaceuticals with 2-pyrrolidinone scaffold.

2-Pyrrolidinones are a class of heterocyclic compounds with a vast diversity of derivatives of biological potential. This structural feature is widely spread in many natural products possessing a large range of biological activities. 2-Pyrrolidinone-based Pyrrocidine A is a known antimicrobial compound produced by endophytic fungi *Sarocladium zeae* [1], (−)-Azaspirene is an angiogenesis inhibitor that is isolated from the fungus *Neosartorya* sp. [2], feeding deterrent Ypaoamide stimulate glucose uptake [3], Salinosporamide A, aquatic natural product, which is created by the constraining aquatic bacteria *Salinispora tropica* and *Salinispora arenicola*, is a potent proteasome inhibitor being studied as a potential anticancer agent [4] and many others. Their significant role is highlighted not only by the wide variety of pharmacological properties [5,6,7,8,9,10] but also by the efficient approved pharmaceutical products such as Cromakalim, which is a potassium channel-opening vasodilator, Nebracetam, known as a nootropic M1-muscarinic agonist, which induces a rise of intracellular Ca^2+^ concentration, a respiratory stimulant Doxapram, as well as Ethosuximide, a medication for prevention and control of the absence or petit mal seizures [11] (Figure 1). Therefore, the synthesis and evaluation of biological properties of compounds containing this structural element remain a very important area of medicinal chemistry for the discovery and development of efficient therapeutic preparations.

As those of 5-oxopyrrolidines, the hydrazones having great biopotential attract more and more researchers involved in the discovery and development of effective pharmaceuticals. The increasing interest in the chemistry of these compounds is related to the fact that such types of compounds are proved to be efficient as an analgesic, anti-inflammatory [12], antiviral [13], antimicrobial, [12,14], antitumor [15], anticonvulsant [16] agents show strong antidepressant [17], cardioprotective [18], and antiplatelet [19] properties as well as demonstrate efficiency as antifungal agents [20]. Furthermore, hydrazones can scavenge free radicals, which are the main culprits in different diseases arising from oxidative stress. That includes cardiovascular and Alzheimer’s diseases, skin cancer, as well as various inflammation, and oxidative damage to proteins and DNA [21,22]. The hydrazone derivatives appeared to possess antioxidant, antiproliferative, and photoprotective activities and are useful for the prevention of skin cancer and helpful in sunscreen formulations [23,24]. The ongoing drug discovery for effective pharmaceutical agents targeting various cancers and infectious disease agents promotes the assessment of a combination of biologically effective moieties in the molecules, one of which is the model of 2-pyrrolidinone and hydrazone fragments. The effect of such a combination is shown to possess antihypertensive [25], antifungal [26], and antibacterial [27] activities. Our extensive studies [28,29,30,31] confirmed that and extended this investigation. The works focused on the synthesis and evaluation of bioefficacy of 2-pyrrolidinone-based hydrazone derivatives and approved them to be a unique structural moiety for the design of agents with an antibacterial, antioxidant, anticancer, and human carbonic anhydrase inhibition activity.

The assessment for their antimicrobial properties against multidrug-resistant Gram-negative (*Klebsiella pneumoniae*, *Stenotrophomonas maltophilia*, *Pseudomonas aeruginosa*) and Gram-positive (*Staphylococcus aureus*) pathogens, and pathogenic fungi (*Candida auris*, *Candida albicans*, *Aspergillus fumigatus*) harboring genetically defined resistance mechanisms revealed bishydrazone with favorable (MIC 2 µg/mL) antibacterial activity against *S. aureus*, which was independent of the existing antimicrobial resistance phenotype and was comparable to the antimicrobial activity of vancomycin and much higher than that of methicillin and cefoxitin. Furthermore, the antifungal properties appeared to be excellent as the hydrazones possessing a 5-oxopyrrolidine structure showed significantly high MIC (0.9–1.9 µg/mL) against *Candida tenuis* VKMY-70 and *Aspergillus niger* VKM F-1119 which surpassed Nystatin (7.8 and 15.6 µg/mL, respectively) the antibiotic used to treat various fungal infections [32].

In this paper, we describe the synthesis of 5-oxopyrrolidine derivatives with the acetamide moiety and characterization of their in vitro antimicrobial and anticancer activity. The choice of the acetamide phenyl moiety, in this case, was determined by the wide variety of biological properties of the compounds bearing this fragment [33,34,35,36] as well as the easy deacetylation possibility to obtain compounds with the free amino group.

## 2. Results and Discussion

### 2.1. Synthesis

**Scheme 1:**
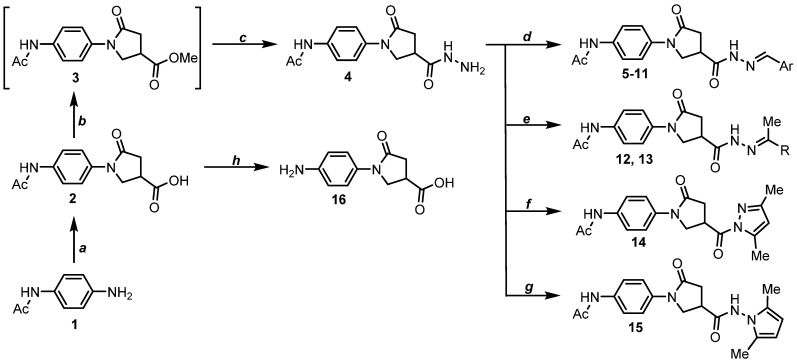
Synthesis of compounds **2**–**16**. **5**, Ar = C_6_H_5_, **6**, Ar = 4-ClC_6_H_4_, **7**, Ar = 4-BrC_6_H_4_, **8**, Ar = 4-Me_2_NC_6_H_4_, **9**, Ar = 4-MeOC_6_H_4_. **10**, Ar = 2,5-di(MeO)C_6_H_3_, **11**, Ar = 2,4,6-tri(MeO)C_6_H_2_, **12**, R = Me, **13**, R = Et. Reagents and conditions: (**a**) itaconic acid, water, Δ, 12 h, 5% HCl, 96%; (**b**,**c**) MeOH, H_2_SO_4_, Δ, 20 h, N_2_H_4_∙H_2_O, Δ, 2 h, 97%; (**d**) water, HCl, ArCHO+2-PrOH, Δ, 2 h, 38–98%; (**e**) acetone or ethyl methyl ketone, Δ, 18 h, 56.2% or 61%; (**f**) 2-PrOH, pentane-2,4-dione, HCl, Δ, 18 h, 30%; (**g**) 2-PrOH, hexane-2,5-dione, AcOH, Δ, 18 h, 34%; (**h**) 10% HCl, Δ, 12 h, AcONa, 74%.

Above all, our research was focused on the synthesis and verification of antimicrobial and anticancer properties of 5-oxopyrrolidine derivatives. For the solution of this aspiration, the *N*-(4-aminophenyl)acetamide (**1**) was chosen and then reacted with an itaconic acid in water at reflux to give 1-(4-acetamidophenyl)-5-oxopyrrolidine-3-carboxylic acid (**2**) (Scheme 1) as an initial compound for further transformations. Compound **2**, when treated with methanol in the presence of a catalytic amount of sulfuric acid in the reaction mixture, afforded methyl ester **3**, which without the separation, was subjected to hydrazinolysis to give acid hydrazide **4**. To synthesize hydrazones, hydrazide **4** was treated with a 1.5-fold excess of the corresponding aromatic aldehyde. The products were obtained in the range of 38–98% yields. The presence of an amide fragment in the molecules of these compounds and the restricted rotation around this bond allowed the formation of the *E*/*Z* conformers [37], which presence is clearly demonstrated by the NMR spectra. The ^1^H NMR spectra of hydrazones **5**–**9** showed that the presence of the unsubstituted or 4-substituted phenyl ring causes the formation of the *Z* and *E* rotamers in the ratio of 65/35, while when di- or trisubstituted phenyl fragment is attached the ratio of conformers gain the values of 70/30 (**10**) and 75/25 (**11**) indicating the growing stability of the *Z-*form.

To compare the biological properties of the products ketones (acetone and ethyl methyl ketone) that were used in analog reactions, which gave propan-2-ylidenehydrazine or butan-2-ylidenehydrazine derivatives **12** and **13**. Their ^1^H NMR spectra showed the presence of conformational and geometric isomers. To interpret their exact structures, detailed and complex spectroscopic studies are required. Whereas this study aimed to synthesize a specific target compound and evaluate its biological properties, a detailed structural analysis was not performed.

The target azoles **14** and **15** were easily obtained from the hydrazide **4** and the appropriate aliphatic diketone. When reacting with pentane-2,4-dione (2,4-PD) in propan-2-ol with the addition of a catalytic amount of hydrochloric acid, the 3,5-dimethylpyrazole **14** was prepared, while the condensation with hexane-2,5-dione (2,5-HD) at the same conditions but using acetic acid as a catalyst instead the 2,5-dimethylpyrrole derivative **15** was formed. The structures were confirmed by their spectral data. In the ^1^H NMR spectrum of **14**, the protons belonging to the CH of the pyrazole cycle gave a singlet at 6.23 ppm, and protons of two methyl groups of the pyrazole ring gave two singlets in up-field of the spectrum, at 2.21 and 2.49 ppm. The ^13^C NMR spectrum showed resonances at 111.59, 13.56, and 14.07 ppm, respectively. A structure of pyrrole **15** was approved by the presence of characteristic peaks at 1.99, 5.65, and 10.90, 10.91 ppm, which were assigned to the protons of the methyl groups, the pyrrole CH, and the amide group. The ^13^C NMR spectrum showed characteristic spectral lines at 10.96 and 103.11 ppm, which were assigned to the carbons of the methyl groups and CH–CH fragment of the pyrrole cycle.

To obtain the compound with the free amino group, the deacylation reaction of the synthesized compound **2** was performed in refluxing dilute hydrochloric acid, followed by transferring the resulting amino hydrochloride into the base form **16** with sodium acetate. The ^1^H NMR spectrum of compound **16** revealed a singlet at 7.65 ppm integrated for 2 protons, which is characteristic of the amine group attached to the phenyl ring in the molecule.

To compare the chemical properties of the compounds, the hydrazide **17** bearing amine, and hydrazide functional groups were prepared. A refluxing mixture of acid **16** and hydrazine monohydrate in toluene gave the target hydrazide **17** over 16 h. When interpreting the ^1^H NMR spectrum of compound **17**, the broad singlets belonging to 2NH_2_ and NH groups, as expected, were at 5.57 and 9.29 ppm, respectively.

**Scheme 2:**
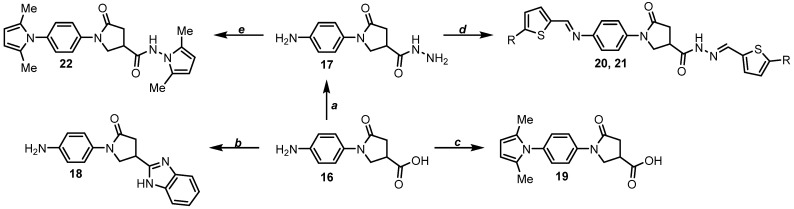
Some chemical transformations of carboxylic acid **16** and acid hydrazide **17**. **20**, R = H; **21**, R = NO_2_. Reagents and conditions: (**a**) toluene, N_2_H_4_∙H_2_O, Δ, 16 h, 83.5%; (**b**) *o*-phenylenediamine, 6N HCl, Δ, 36 h, AcONa, 75%; (**c**) 2-PrOH, hexane-2,5-dione, AcOH, Δ, 4 h, 57%; (**d**) water, HCl, the corresponding thiophene-2-carbaldehyde+2-PrOH, Δ, 2 h, 57.3% (**20**) or 12 h, 66.8% (**21**); (**e**) MeOH, hexane-2,5-dione, AcOH, Δ, 4 h, 32%.

The functional groups present in compounds **16** and **17** were applied to obtain variously substituted derivatives **18**–**22** (Scheme 2). Thus, acid **16** was condensed with *o*-phenylenediamine in refluxing diluted hydrochloric acid (1:1) for 36 h. The target benzimidazole **18** was separated by the addition of sodium acetate to an aqueous solution of the formed precipitate. The NMR spectra showed good agreement with the expected structure.

Then, carboxylic acid **16** was reacted with a 4-fold excess of hexane-2,5-dione and a catalytic amount of acetic acid in propan-2-ol at reflux for 4 h. The reaction yielded 2,5-dimethylpyrrole derivative **19**. The formed pyrrole ring was approved by the intense singlets at 1.95 (2CH_3_) and 5.78 (CH–CH_pyr_) ppm in the ^1^H NMR spectrum. The ^13^C NMR spectrum showed peaks at 12.86 and 105.79 ppm, respectively, which are characteristic of the 2,5-dimethylpyrrole cycle.

To verify the hydrazide **17** and to evaluate the impact of substituents on the biological properties of compounds, condensation reactions with carbonyl compounds (heteroaromatic aldehydes and aliphatic diketone) were carried out. The reactions with 2-thiophenecarboxaldehyde and its 5-nitro analog in an aqueous propanolic (15:1) medium in the presence of hydrochloric acid as a catalyst leading to the formation of the appropriate hydrazones **20** and **21**. The ^1^H NMR spectra of these compounds exhibited four down-field singlet signals at 11.55, 11.57, 11.59, and 11.61 (**20**) and two ones at 11.99 and 12.02 (**21**) ppm attributed to the protons of the NH. The azomethine CH=N protons resonated between 8.20–8.85 (**20**) and 8.47–9.01 (**21**) ppm.

Compound **22** bearing two 2,5-dimethylpyrrole cycles was prepared from compound **17** by the condensation reaction with a 4-fold excess of hexane-2,5-dione in methanol with the addition of a catalytic amount of glacial acetic acid. The reaction at reflux for 4 h resulted in the formation of a desired product **22**. The presence of amine and hydrazide groups afforded an asymmetric bis(pyrrole) molecule. The ^1^H NMR spectrum fully confirmed the formed structure: two singlets at 1.96 and 2.01 ppm each integrated for six protons were attributed for 4CH_3_, and two singlets at 5.65 and 5.79 ppm where each integrated for two protons were ascribed to 2CH–CH_pyr_ and only one signal at 10.94 ppm was assigned to NH exhibiting that one pyrrole is attached directly to the phenyl ring, and another one is inserted in the molecule via amide moiety.

### 2.2. The Anticancer Activity of 5-Oxopyrrolidine Derivatives ***2*** and ***4**–**22***

To characterize the biological activity of compounds **2** and **4**–**22**, we determined the anticancer properties of novel 5-oxopyrrolidine derivatives using well established A549 human lung adenocarcinoma model. To better understand the toxicity of the novel compounds, we also used HSAEC-1 KT human small airway epithelial cells that served as non-cancerous cells derived from the pulmonary environment. We exposed the A549 and HSAEC1-KT cells with a fixed 100 µM concentration of each compound for 24 h and evaluated the post-treatment viability using an MTT assay. The compound-mediated cytotoxicity was compared with cisplatin (CP), a standard chemotherapeutic drug used for lung cancer treatment.

**Figure 2:**
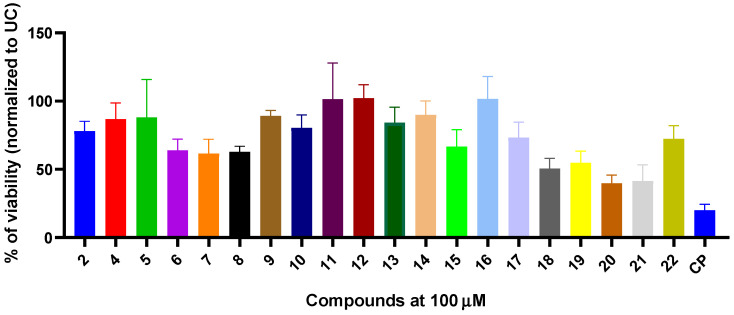
The anticancer activity of 5-oxopyrrolidine derivatives **2** and **4**–**22** on A549 human lung adenocarcinoma cells. The cells were exposed to 100 µM of each compound or cisplatin (CP) for 24 h and post-treatment viability was calculated using untreated control as a normalization marker. Data are shown as mean ∓ SD from 3 experimental replicas.

**Figure 3:**
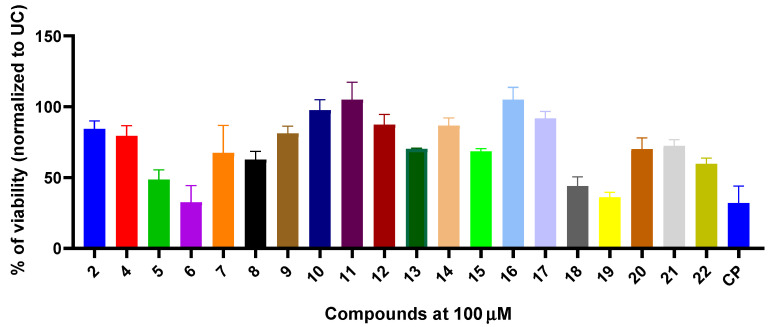
The in vitro cytotoxic activity of 5-oxopyrrolidine derivatives **2** and **4**–**22** on non-cancerous HAEC1-KT human small airway epithelial cells. The cells were exposed to 100 µM of each compound or cisplatin (CP) for 24 h and post-treatment viability was calculated using untreated control as a normalization marker. Data are shown as mean ∓ SD from 3 experimental replicas.

The compounds exhibited the structure-depended anticancer activity on A549 cells. Carboxylic acid **2** that was generated from starting compound **1** showed weak anticancer activity and resulted in 78–86% post-treatment viability (Figure 2). The compound conversion to acid hydrazide **4** did not enhance the anticancer activity. Notably, the conversion of hydrazide **4** to hydrazone greatly improved the anticancer activity in a structure-dependent manner (Figure 2). The incorporation of phenyl ring (compound **5**) did not significantly affect the anticancer activity in comparison to compound **4**. Furthermore, 4-chlorophenyl and 4-bromophenyl substitutions (**6** and **7**, respectively) enhanced the anticancer activity by reducing the A549 viability to 64 and 61%, respectively. Furthermore, besides enhanced anticancer activity, compound **6** exhibited increased cytotoxicity towards non-cancerous HSAEC1-KT cells (Figure 3). Interestingly, 4-dimethylamino phenyl substitution showed the most potent anticancer activity (**8**), which was significantly higher than compound **4** (*p* < 0.05), while incorporation 4-methoxy group in the phenyl ring ameliorated the anticancer activity (compound **9**). Compounds **6** and **7** reduced the A549 viability, although no statistically significant effect was observed when the anticancer activity of **6** and **7** were compared with starting compound **4**. Furthermore, di- and trimethoxy substitutions in the phenyl ring (compounds **10** and **11**) resulted in a significant loss of anticancer activity (*p* < 0.05) in comparison to compound **8** (Figure 2).

Hydrazones **12** and **13** showed weak to no anticancer activity on A549 cells as well as weak cytotoxic activity on HSAEC1-KT cells (Figure 3).

Among azole derivatives, 2,5-dimethylpyrrole derivative **15** exerted more potent activity than 3,5-dimethylpyrazole **14** by reducing the A549 viability to 66%. Compound **15** exhibited noticeable cytotoxic activity towards non-cancerous cells, suggesting that the 2,5-dimethylpyrrole moiety is important for conferring the cytotoxic activity in normal and cancerous cells. Compounds containing free amino group, except **16**, and their derivatives bearing various structural substitutions showed more potent anticancer activity than those with an acetylamino fragment in the molecules with no significant cytotoxic effect on non-cancerous cells (Figure 2 and Figure 3). Bis hydrazone **20**, containing 2-thienyl fragments and its analog **21** with two 5-nitrothienyl moieties in the structure, demonstrated the highest anticancer activity amongst all tested 5-oxopyrrolidine derivatives and had favorable low cytotoxic properties on non-cancerous cells (Figure 2 and Figure 3).

Finally, the structure–activity relationship study of the investigated hydrazones **5**–**11**, **20**, **21** has shown (Figure 2) that the anticancer activity of the hydrazones **5**–**11** with aromatic moieties is lower compared to hydrazones **20**, **21** containing heterocyclic fragments. The most active compounds of aromatic hydrazones were compounds containing dimethylamino-, chloro-, and bromo- substituents in the aromatic ring. As can be seen from the study data, compound **16** exhibited low anticancer activity, but when its functional groups are modified to fragments of benzimidazole (**18**) or dimethylpyrrole (**19**), their anticancer activity increases strongly. Therefore, in the future, it would be worth expanding such modifications in search of new compounds with high anticancer activity.

These results suggest that the 5-oxopyrrolidine derivatives can suppress the viability in the A549 human lung cell adenocarcinoma model in a structure-depended manner. In addition to that, 5-oxopyrrolidine derivatives obtained from compounds containing the free amino group exert the most promising anticancer activity demonstrating the importance of the free amino group in the search for anticancer agents with low cytotoxicity toward non-cancerous cells.

### 2.3. The Antimicrobial Activity of 5-Oxopyrrolidine Derivatives ***2*** and ***4**–**22***

After characterizing the anticancer activity of compounds **2** and **4**–**22,** we further explored their antimicrobial activity using multidrug-resistant and clinically significant pathogens. The compounds **2** and **4**–**22** were screened using carbapenemases producing *Enterobacteriales* (*Klebsiella pneumoniae*, *Escherichia coli*), multidrug-resistant *Pseudomonas aeruginosa*, carbapenems, and polymyxin-resistant *Acinetobacter baumannii* and methicillin-resistant and vancomycin-intermediate *Staphylococcus aureus* strains.

The compounds **2** and **4**–**22** showed no antimicrobial activity when screened against Gram-negative pathogens (MIC > 64 µg/mL) (Supplementary Table S1). Interestingly, compound **21** bearing nitro substitution demonstrated promising antimicrobial activity against *Staphylococcus aureus* TCH 1516 (USA 300) strain (MIC 2 µg/mL), demonstrating the Gram-positive bacteria-directed activity (Supplementary Table S1).

After demonstrating that compound **21** exerts the in vitro antibacterial activity against *S. aureus*, we decided to check whether the *S. aureus*-directed activity depends on the pre-existing *S. aureus* resistance mechanisms. We have screened compound **21** using vancomycin-intermediate and oxazolidines (linezolid/tedizolid) resistant strains and compared the MIC values with clinically approved drugs.

The compound **21** demonstrated favorable activity (MIC 1–8 µg/mL) against multidrug-resistant and vancomycin-intermediate *Staphylococcus aureus* isolates harboring major multidrug-resistance determining mechanisms (Table 1). On the other hand, higher MIC values (4–64 µg/mL) were observed when linezolid/tedizolid-resistant strains were used for the assays (Table 2).

Many nitro groups containing compounds have enhanced antimicrobial activity under anaerobic conditions. As an example, FDA approved drug metronidazole has excellent activity against anaerobic bacteria, while little to no activity against aerobes. After demonstrating that the nitro group containing compound **21** shows favorable activity against Gram-positive pathogens, we evaluated if the nitro group could confer enhanced activity against anaerobes. To do so, we used representative anaerobic pathogens and determined the MIC for the compound **21** and metronidazole, which served as a controlled drug (Table 3). Compound **21** demonstrated higher antimicrobial activity against Gram-positive pathogens (*C. difficile* and *C. perfringens*). The weak activity was also observed against Gram-negative pathogens, suggesting that under anaerobic conditions, compound **21** could confer some weak activity against Gram-negative anaerobic bacteria. Compound **21** did not exhibit greater activity than metronidazole, which was used as a control agent.

Collectively, these results demonstrate that 5-oxopyrrolidine derivative **21** shows promising and selective antimicrobial activity against Gram-positive pathogens with the highest activity against multidrug-resistant *S. aureus* with genetically defined and emerging resistance profiles. The 5-oxopyrrolidine derivatives could be potentially explored as promising pharmacophores for a further hit to lead development as antimicrobial candidates targeting challenging resistance mechanisms in the high priority pathogens.

## 3. Materials and Methods

### 3.1. Synthesis

Reagents, antibiotics, and solvents were obtained from Sigma-Aldrich (St. Louis, MO, USA) and used without further purification. The reaction course and purity of the synthesized compounds were monitored by TLC using aluminum plates precoated with Silica gel with F254 nm (Merck KGaA, Darmstadt, Germany). Melting points were determined with a B-540 melting point analyzer (Büchi Corporation, New Castle, DE, USA) and were uncorrected. NMR spectra were recorded on a Brucker Avance III (400, 101 MHz) spectrometer (Bruker BioSpin AG, Fällanden, Switzerland). Chemical shifts were reported in (*d*) ppm relative to tetramethylsilane (TMS) with the residual solvent as internal reference (DMSO-d_6_, *d* = 2.50 ppm for 1H and *d* = 39.5 ppm for ^13^C). Data were reported as follows: chemical shift, multiplicity, coupling constant (Hz), integration, and assignment. IR spectra (ν, cm^−1^) were recorded on a Perkin–Elmer Spectrum BX FT–IR spectrometer (Perkin–Elmer Inc., Waltham, MA, USA) using KBr pellets. Elemental analyses (C, H, N) were conducted using the Elemental Analyzer CE-440 (Exeter Analytical, Inc., Chelmsford, MA, USA); their results were found to be in good agreement (±0.3%) with the calculated values.

*1-(4-Acetamidophenyl)-5-oxopyrrolidin-3-carboxylic acid* (**2**). A mixture of acetamide **1** (75 g, 0.5 mol), itaconic acid (98 g, 0.75 mol,) and water (100 mL) was refluxed for 12 h, then 5% hydrochloric acid (100 mL) was added to the mixture was stirred for 5 min. After cooling the mixture, the formed crystalline solid was filtered off, washed with water, and purified by dissolving it in 5% sodium hydroxide solution, filtering and acidifying the filtrate with hydrochloric acid to pH 5 to give the title compound **2** (white solid, yield 126.1 g, 96%, m. p. 237–238 °C).

^1^H NMR (400 MHz, DMSO–*d_6_*) δ: 2.03 (s, 3H, CH_3_), 2.65–2.77 (m, 2H, CH_2_CO), 3.32–3.38 (m, 1H, CH), 3.92–4.01 (m, 2H, NCH_2_), 7.56 (s, 4H, H_Ar_), 9.93 (s, 1H, NH), 12.77 (s, 1H, OH) ppm.

^13^C NMR (101 MHz, DMSO–*d_6_*) δ, m. d.: 23.95 (CH_3_), 35.12, 35.14 (CH, CH_2_CO), 49.96, 50.03 (NCH_2_), 119.17, 119.71, 119.94, 134.33, 135.27, 135.63 (C_Ar_); 168.12, 171.43, 174.26 (C=O) ppm.

IR (KBr): v_max_ 3454–2536 (NH+OH); 1727; 1677; 1644 (C=O); 1512 (C=N); 1172 (C-N) cm^−1^.

Calcd. for C_13_H_14_N_2_O_4_, %: C 59.54; H 5.38; N 10.68; Found, %: C 59.83; H 5.42; N 10.42.

*N-(4-(4-(hydrazinecarbonyl)-2-oxopyrrolidin-1-yl)phenyl)acetamide* (**4**). A mixture of carboxylic acid **2** (7.87 g, 0.03 mol), methanol (100 mL), and sulfuric acid (10 drops) were refluxed for 20 h, then hydrazine monohydrate was added (12 g, 0.24 mol), and the reaction mixture was heated at reflux for 2 h. After completion of the reaction, the mixture was cooled down, and the formed precipitate was filtered off, washed with propan-2-ol, and diethyl ether to give the title compound **4** (white solid, yield 8.25 g, 97%, m. p. 221–222 °C (from water).

^1^H NMR (400 MHz, DMSO–*d_6_*) δ: 2.02 (s, 3H, CH_3_), 2.60–2.71 (m, 2H, CH_2_CO), 3.11–3.19 (m, 1H, CH), 3.75–3.91 (m, 2H, NCH_2_), 4.41 (br. s. 2H, NH_2_), 7.55 (s, 4H, H_Ar_), 9.27 (s, 1H, N*H*NH_2_), 9.93 (s, H, NHCO) ppm.

^13^C NMR (101 MHz, DMSO–*d_6_*) δ: 23.94 (CH_3_), 34.08, 35.65 (CH, CH_2_CO), 50.76 (NCH_2_), 119.17, 119.85, 134.37, 135.55 (C_Ar_), 168.12, 171.59, 171.69 (C=O) ppm.

IR (KBr): v_max_ 3367–3220(NH+NH_2_); 1673; 1645; 1604 (C=O); 1108 (C-N) cm^−1^.

Calcd. for C_13_H_16_N_4_O_3_, %: C 56.51; H 5.84; N 20.28. Found, %: 56.77; H 5.70; N 19.99.

*General procedure for the preparation of hydrazones* **5**–**11**. To a hot solution of hydrazide **4** (0.5 g, 1.8 mmol) in water (60 mL) with the addition of hydrochloric acid (5 drops), the solution of the corresponding aromatic aldehyde (2.7 mmol) in propan-2-ol (5 mL) was added, and the mixture was heated at reflux for 2 h, then cooled down. The obtained solid was filtered off, washed with water, and dried to give the title compounds **5–11**.

*N-(4-(4-(2-benzylidenehydrazine-1-carbonyl)-2-oxopyrrolidin-1-yl)phenyl)acetamide* (**5**).

White solid yield 0.53 g, 80.8%, mp 203–204 °C (from 1,4-dioxane).

^1^H NMR (400 MHz, DMSO–*d_6_*) δ: (*Z/E* 65/35) 2.02 (s, 3H, CH_3_), 2.74–2.82 (m, 2H, CH_2_CO), 3.91–4.13 (m, 3H, CH, NCH_2_), 7.41–7.46 (m, 3H, H_Ar_), 7.57 (s, 4H, H_Ar_), 7.67–7.72 (m, 2H, H_Ar_), 8.04, 8.22 (2s, 1H, N=CH), 9.93, 9.94 (2s, 1H, NHCO), 11.57, 11.63 (2 s, 1H, NH) ppm.

IR (KBr): v_max_ 3294; 3330 (NH); 1672; 1658; 1579 (C=O); 1515 (C=N); 1172 (C-N) cm^−1^.

Calcd. for C_20_H_20_N_4_O_3_, %: C 65.92; H 5.53; N 15.38. Found, %: 66.00; H 5.53; N 15.32.

*N-(4-(4-(2-(4-chlorobenzylidene)hydrazine-1-carbonyl)-2-oxopyrrolidin-1-yl)phenyl)acetamide* (**6**).

White solid, yield 0.65 g, 90.8%, mp 213–214 °C (from 1,4-dioxane).

^1^H NMR (400 MHz, DMSO–*d_6_*) δ: (*Z/E* 65/35) 2.02 (s, 3H, CH_3_), 2.69–2.81 (m, 2H, CH_2_CO), 3.89–4.11 (m, 3H, CH, NCH_2_), 7.50 (d, *J* = 8.3 Hz, 2H, H_Ar_), 7.57 (s, 4H, H_Ar_), 7.71–7.75 (m, 2H, H_Ar_), 8.02, 8.21 (2s, 1H, N=CH), 9.93 (s, 1H, NHCO), 11.63, 11.71 (2 s, 1H, NH) ppm.

^13^C NMR (101 MHz, DMSO–*d_6_*) δ: 23.93 (CH_3_), 32.81, 34.83, 35.55 (CH, CH_2_CO), 50.10, 50.54 (NCH_2_), 119.16, 119.89, 119.96, 128.55, 128.72, 128.91, 133.08, 134.32, 135.58, 142.39, 145.72, 168.09, 168.81, 171.46, 171.66, 173.67 (C_Ar_, CH=N, C=O) ppm.

IR (KBr): v_max_ 3238; 3342 (NH); 1672; 1602; 1582 (C=O); 1515 (C=N); 1133 (C-N) cm^−1^.

Calcd. for C_20_H_19_ClN_4_O_3_, %: C 60.23; H 4.80; N 14.05. Found, %: C 59.97; H 4.70; N 13.97.

*N-(4-(4-(2-(4-bromobenzylidene)hydrazine-1-carbonyl)-2-oxopyrrolidin-1-yl)phenyl)acetamide* (**7**).

White solid, yield 0.78 g, 98%, mp 224–225 °C (from 1,4-dioxane).

^1^H NMR (400 MHz, DMSO–*d_6_*) δ: (*Z/E* 65/35) 2.02 (s, 3H, CH_3_), 2.67–2.86 (m, 2H, CH_2_CO), 3.88–4.19 (m, 3H, CH, NCH_2_), 7.46–7.82 (m, 8H, H_Ar_), 8.01, 8.19 (2s, 1H, N=CH), 9.93, 9.94 (2s, 1H, NHCO), 11.63, 11.70 (2s, 1H, NH) ppm.

IR (KBr): v_max_ 3233; 3198 (NH); 1670; 1655; 1646 (C=O); 1515 (C=N); 1304 (C-N) cm^−1^.

Calcd. for C_20_H_19_BrN_4_O_3_, %: C 54.19; H 4.32; N 12.64. Found, %: C 53.94; H 4.45; N 12.44.

*N-(4-(4-(2-(4-(dimethylamino)benzylidene)hydrazine-1-carbonyl)-2-oxopyrrolidin-1-yl)phenyl)acetamide* (**8**).

White solid, yield 0.38 g, 51.8%, mp 174–175 °C (from 1,4-dioxane).

^1^H NMR (400 MHz, DMSO–*d_6_*) δ: (*Z/E* 65/35) 2.02 (s, 3H, CH_3_), 2.69–2.78 (m, 2H, CH_2_CO), 2.95, 2.96 (2s, 6H, N(CH_3_)_2_), 3.91–4.11 (m, 3H, CH, NCH_2_), 6.72, 6.74 (2d, *J* = 6.1 Hz, 2H, H_Ar_), 7.50 (d, *J* = 8.6 Hz, 2H, H_Ar_), 7.57 (s, 4H, H_Ar_), 7.90, 8.06 (2s, 1H, N=CH), 9.93 (s, 1H, NHCO), 11.27, 11.32 (2 s, 1H, NH) ppm.

IR (KBr): v_max_ 3012; 2887 (NH); 1701; 1693; 1655 (C=O); 1517 (C=N); 1124; 1110 (C-N) cm^−1^.

Calcd. for C_22_H_25_N_5_O_3_, %: C 64.85; H 6.18; N 17.19. Found, %: C 64.70; H 6.08; N 16.59.

*N-(4-(4-(2-(4-methoxybenzylidene)hydrazine-1-carbonyl)-2-oxopyrrolidin-1-yl)phenyl)acetamide* (**9**).

White solid, yield 0.42 g, 59.2%, mp 234–235 °C (from 1,4-dioxane).

^1^H NMR (400 MHz, DMSO–*d_6_*) δ: (*Z/E* 65/35) 2.02 (s, 3H, CH_3_), 2.69–2.81 (m, 2H, CH_2_CO), 3.79 (s, 3H, OCH_3_), 3.84–4.16 (m, 3H, CH, NCH_2_), 7.00 (t, *J* = 7.7 Hz, 2H, H_Ar_), 7.36–7.86 (m, 6H, H_Ar_), 7.98, 8.15 (2s, 1H, N=CH), 9.93(s, 1H, NHCO), 11.44, 11.50 (2s, 1H, NH) ppm.

IR (KBr): v_max_ 3026; 3113 (NH); 1675; 1671; 1688 (C=O); 1511 (C=N); 1231(C-N) cm^−1^.

Calcd. for C_21_H_22_N_4_O_4_, %: C 63.95; H 5.62; N 14.20. Found, %: C 63.79; H 5.56; N 14.48.

*N-(4-(4-(2-(2,5-dimethoxybenzylidene)hydrazine-1-carbonyl)-2-oxopyrrolidin-1-yl)phenyl)acetamide* (**10**).

White solid, yield 0.74 g, 97.4%, mp 190–191 °C (from 1,4-dioxane).

^1^H NMR (400 MHz, DMSO–*d_6_*) δ: (*Z/E* 70/30) 2.02, 2.03 (2s, 3H, CH_3_), 2.71–2.80 (m, 2H, CH_2_CO), 3.72, 3.73 (2s, 3H, OCH_3_), 3.79, 3.81 (2s, 3H, OCH_3_), 3.93–4.13 (m, 3H, CH, NCH_2_), 6.97–7.06 (m, 2H, H_Ar_), 7.30, 7.35 (2d, *J* = 3.1 Hz, 1H, H_Ar_), 7.53–7.60 (m, 4H, H_Ar_), 8.33, 8.53 (2s, 1H, N=CH), 9.92, 9.94 (2s, 1H, NHCO), 11.53, 11.64 (2s, 1H, NH) ppm.

IR (KBr): v_max_ 3113; 3250 (NH); 1708; 1689; 1671 (C=O); 1494 (C=N); 1226 (C-N) cm^−1^.

Calcd. for C_22_H_24_N_4_O_5_, %: C 62.25; H 5.70; N 13.20. Found, %: C 62.21; H 5.76; N 12.96.

*N-(4-(2-oxo-4-(2-(2,4,6-trimethoxybenzylidene)hydrazine-1-carbonyl)pyrrolidin-1-yl)phenyl)acetamide* (**11**).

White solid, yield 0.31 g, 38%, mp 194–195 °C (from 1,4-dioxane).

^1^H NMR (400 MHz, DMSO–*d_6_*) δ: (*Z/E* 75/25) 2.02 (s, 3H, CH_3_), 2.58–2.81 (m, 2H, CH_2_CO), 3.78, 3.82 (2s, 9H, 3OCH_3_), 3.89–4.13 (m, 3H, NCH_2_, CH), 6.27 (s, 2H, H_Ar_), 7.56 (s, 4H, H_Ar_), 8.19, 8,33 (2s, 1H, N=CH), 9.93 (s, 1H, NHCO), 11,18, 11.25 (2s, 1H, NH) ppm.

IR (KBr): v_max_ 3332; 3478 (NH); 1691; 1672; 1658 (C=O); 1565 (C=N); 1132 (C-N) cm^−1^.

Calcd. for C_23_H_26_N_4_O_6_, %: C 60.78; H 5.77; N 12.33. Found, %: C 60.97; H 5.66; N 12.12.

*General procedure for the preparation of hydrazones* **12**, **13**. A mixture of hydrazide **4** (0.5 g, 1.8 mmol) and acetone (**12**) or ethyl methyl ketone (**13**) (15 mL) was heated at reflux for 18 h, then cooled down. The obtained solid was filtered off, washed with acetone, and dried to give the title compound **12** (white solid, yield 0.32 g, 56.2%, mp 186–187 °C (from acetone) or compound **13** (white solid, yield 0.36 g, 61%, mp 195–196 °C (from acetone).

*N-(4-(2-oxo-4-(2-(propan-2-ylidene)hydrazine-1-carbonyl)pyrrolidin-1-yl)phenyl)acetamide* (**12**).

^1^H NMR (400 MHz, DMSO–*d_6_*) δ: 1.87, 1.88 (s, 3H, CH_3_), 1.93 (s, 3H, CH_3_), 2.02 (s, 3H, CH_3_), 2.57–2.79 (m, 2H, CH_2_CO), 3.35–3.46 (m, 0.65H, CH), 3.81–4.06 (m, 2H, NCH_2_, 0.35H, CH), 7.55 (s, 4H, H_Ar_), 9.92 (s, 1H, NHCO), 10.21, 10.22, 10.30, 10.31(4s, 1H, NH) ppm.

IR (KBr): v_max_ 3183; 3343 (NH); 1701; 1656; 1607; (C=O); 1596 (C=N); 1221 (C-N) cm^−1^.

Calcd. for C_16_H_20_N_4_O_3_, %: C 60.75; H 6.37; N 17.71. Found, %: C 60.51; H 6.14; N 17.60.

*N-(4-(4-(2-(butan-2-ylidene)hydrazine-1-carbonyl)-2-oxopyrrolidin-1-yl)phenyl)acetamide* (**13**).

^1^H NMR (400 MHz, DMSO–*d_6_*) δ: (*Z/E* 65/35) 0.87–1.09 (m, 3H, CH_3_CH_2_), 1.85, 1.87, 1.91, 1.93 (4s, 3H, CH_3_), 2.02 (s, 3H, CH_3_), 2.17–2.35 (m, 2H, CH_2_CO), 3.37–3.47 (m, 0.65H, CH), 3.83–4.04 (m, 2H, CH_2_CO, 0.35H, CH), 7.56 (s, 4H, H_Ar_), 9.92 (2s, H, NHCO), 10.19, 10.21, 10.25, 10.30, 10.33, 10.40 ppm.

IR (KBr): v_max_ 3165; 3241 (NH); 1655; 1598; 1574 (C=O); 1516 (C=N); 1129 (C-N) cm^−1^.

Calcd. for C_17_H_22_N_4_O_3_, %: C 61.80; H 6.71; N 16.96; Found, %: C 61.96; H 6.60; N 17.06.

*N-(4-(4-(3,5-dimethyl-1H-pyrazol-1-carbonyl)-2-oxopyrrolidin-1-yl)phenyl)acetamide* (**14**). To a solution of hydrazide **4** (0.5 g, 1.8 mol) in propan-2-ol (50 mL), pentane-2,4-dione (0.55 mL, 5.4 mmol), and hydrochloric acid (5 drops) were added and the mixture was heated at reflux for 18 h, then cooled down. The formed precipitate was filtered off and washed with diethyl ether to give the title compound **14** (white solid, yield 0.18 g, 30%, mp 174–175 °C (from propan-2-ol).

^1^H NMR (400 MHz, DMSO–*d_6_*) δ: 2.02 (s, 3H, CH_3_), 2.21, 2.49 (2s, 6H, 2CH_3_), 2.81–2.91 (m, 2H, CH_2_CO); 3.97–4.03 (m, 1H, NCH_2_), 4.15–4.22 (m, 1H, NCH_2_), 4.43–4.50 (m, 1H, CH), 6.23 (m, 1H, CH), 7.56 (s, 4H, H_Ar_), 9.93, 10.20 (2s, 1H, NHCO) ppm.

^13^C NMR (101 MHz, DMSO–*d_6_*) δ: 13.56, 14.07 (2CH_3_), 23.93 (CH_3_), 35.02, 35.37 (CH, CH_2_CO), 50.22 (NCH_2_), 111.59, 119.13, 120.06, 134.20, 135.69, 143.89, 152.14 (C_Ar_), 168.09, 171.18, 172.65 (3C=O) ppm.

IR (KBr): v_max_ 3340; 3267 (NH); 1728; 1678; 1693 (C=O); 1519 (C=N); 1309; 1285; 1225 (C-N) cm^−1^.

Calcd. for C_18_H_20_N_4_O_3_, %: C 63.52; H 5.92; N 16.46. Found, %: C 63.42; H 5.83; N 16.69.

*N-(4-(4-(2-(2,5-dimethyl-1H-pyrrol-1-yl)acetyl)-2-oxopyrrolidin-1-yl)phenyl)acetamide* (**15**). To a solution of hydrazide **4** (0.5 g, 1.8 mol) in propan-2-ol (50 mL), hexane-2,5-dione (0.63 mL, 5.4 mmol), and acetic acid (5 drops) were added and the mixture was heated at reflux for 18 h and then cooled down. The formed precipitate was filtered off and washed with diethyl ether to give the title compound **15** (white solid, yield 0.22 g, 34%, mp 225–226 °C (from propan-2-ol).

^1^H NMR (400 MHz, DMSO–*d_6_*) δ: 1.99 (s, 6H, 2CH_3_), 2.02 (s, 3H, CH_3_), 2.67–2.91 (m, 2H, CH_2_CO), 3.42–3.47 (m, 1H, CH), 3.92–4.16 (m, 2H, NCH_2_), 5.65 (m, 2H CH-CH), 7.57 (s, 4H, H_Ar_), 9.94 (s, 1H, NHCO), 10.90, 10.91 (s, H, NH) ppm.

^13^C NMR (101 MHz, DMSO–*d_6_*) δ: 10.96 (2CH_3_), 23.94 (CH_3_), 34.07, 35.56 (CH, CH_2_CO), 50.48 (NCH_2_), 103.11, 119.19, 120.02, 126.75, 134.27, 135.69 (C_Ar_), 168.13, 171.25, 171.93 (3C=O) ppm.

IR (KBr): v_max_ 3261; 3333 (NH); 1682; 1597; 1527 (C=O); 1143; 1130; 1111 (C-N) cm^−1^.

Calcd. for C_19_H_22_N_4_O_3_, %: C 64.39; H 6.26; N 15.81. Found, %: C 63.58; H 5.43; N 15.62.

*1-(4-Aminophenyl)-5-oxopyrrolidine-3-carboxylic acid* (**16**). To a refluxing 10% aqueous hydrochloric acid solution (100 mL) carboxylic acid **2** (7.87 g, 30 mmol) was added and the mixture was heated at reflux for 12 h, filtered off while hot and sodium acetate (16.4 g, 0.2 mol) was added to the filtrate. The formed solid was filtered off, washed with water, and purified by dissolving it in an aqueous 5% sodium carbonate solution, filtering and acidifying the filtrate with acetic acid to pH 6 to give the title compound **16** (white solid, yield 4.89 g, 74%, mp 209–210 °C.

^1^H NMR (400 MHz, DMSO–*d_6_*) δ: 2.56–2.73 (m, 2H, CH_2_CO), 3.23–3.32 (m, 1H, CH), 3.82–3.96 (m, 2H, NCH_2_), 6.55 (d, *J* = 8.8 Hz, 2H, H_Ar_), 7.22 (d, *J* = 8.8 Hz, 2H, H_Ar_), 7.65 (br. s, 2H, NH_2_) ppm.

^13^C NMR (101 MHz, DMSO–*d_6_*) δ: 34.97, 35.20, 35.30 (CH, CH_2_CO), 50.61 (NCH_2_), 113.67, 121.67, 128.26, 145.79 (C_Ar_), 170.73, 174.48 (2C=O) ppm.

IR (KBr): v_max_ 3345–3267 (NH_2_+OH); 1665; 1625 (C=O); 1171 (C-N) cm^−1^.

Calcd. for C_11_H_12_N_2_O_3_, %: C 59.99; H 5.49; N 12.72. Found, %: C 60.25; H 5.56; N 12.84.

*1-(4-Aminophenyl)-5-oxopyrrolidine-3-carbohydrazide* (**17**). To a solution of compound **16** (11.01 g, 50 mmol) in toluene (200 mL), hydrazine monohydrate (7.5 g, 150 mmol) was added, and the mixture was refluxed for 16 h. After completion of the reaction, the mixture was cooled down, and the formed precipitate was filtered off, washed with propan-2-ol to give the title compound **17** (white solid, yield 9.78 g, 83.5%, mp 214–215 °C (from propan-2-ol).

^1^H NMR (400 MHz, DMSO–*d_6_*) δ: 2.51–2.65 (m, 2H, CH_2_CO), 3.05–3.19 (m, 1H, CH), 3.65–3.90 (m, 2H, NCH_2_), 5.57 (br. s, 4H, 2NH_2_), 6.55 (d, *J =* 8.5 Hz, 2H, H_Ar_), 7.22 (d, *J =* 8.5 Hz, 2H, H_Ar_), 9.29 (br. s, 1H, NH) ppm.

^13^C NMR (101 MHz, DMSO–*d_6_*) δ: 34.20, 35.42 (CH, CH_2_CO), 50.24 (NCH_2_), 113.75, 121.56, 121.59, 128.37, 145.59 (C_Ar_), 168.34, 170.92, 171.72 (C=O) ppm.

IR (KBr): v_max_ 2553; 2654; 2888 (NH+ NH_2_); 1725 (C=O); 1567; 1512 (C=N); 1265 (C-N) cm^−1^.

Calcd. for C_11_H_14_N_4_O_2_, %: C 56.40; H 6.02; N 23.92. Found, %: C 56.30; H 6.32; N 23.82.

*1-(4-Aminophenyl)-4-(1H-benzo[d]imidazol-2-yl)pyrrolidin-2-one* (**18**). A solution of compound **16** (4.4 g, 20 mmol) and *o*-phenylenediamine (4.32 g, 40 mmol) in dilute hydrochloric acid (1:1, 20 mL) was heated at reflux for 36 h, then cooled down. The obtained precipitate was filtered off, washed with water, and dissolved in boiling water (20 mL). Then sodium acetate was added (0.5 g) under stirring. The formed solid was filtered off and washed with water to obtain the title compound **18** (white solid, yield 4.38 g, 75%, mp 356 °C (decomp., from dioxane).

^1^H NMR (400 MHz, DMSO–*d_6_*) δ: 2.92–3.04 (m, 2H, CH_2_CO), 3.98–4.05 (m, 1H, CH), 4.18–4.32 (m, 2H, NCH_2_), 5.13 (br. s, 2H, NH_2_), 6.56 (d, *J =* 8.4 Hz, 1H, H_Ar_), 7.15 (dd, *J* = 6.2, 3.2 Hz, 2H, H_Ar_), 7.27 (d, *J =* 8.4 Hz, 1H, H_Ar_), 7.49–7.54 (m, 2H, H_Ar_), 7.71 (s, 2H, H_Ar_), 12.45 (s, 1H, NH) ppm.

^13^C NMR (101 MHz, DMSO–*d_6_*) δ: 30.65, 30.80, 37.24, 37.48 (CH, CH_2_CO), 50.17, 52.74 (NCH_2_), 113.67, 119.71, 121.63, 128.38, 135.36, 145.71, 154.99, 155.20 (C_Ar_), 170.88, 171.83 (C=O) ppm.

IR (KBr): v_max_ 2640; 2850 (NH), 1659 (C=O), 1518 (C=N), 1181 (C-N) cm^−1^.

Calcd. for C_17_H_16_N_4_O, %: C 69.85; H 5.52; N 19.17. Found, %: C 69.67; H 5.48; N 17.29.

*1-(4-(2,5-Dimethyl-1H-pyrrol-1-yl)phenyl)-5-oxpyrrolidine-3-carboxylic acid* (**19**). To a solution of compound **16** (0.55 g, 2.5 mmol) in propan-2-ol (50 mL) hexane-2,5-dione (1.14 g, 10 mmol) and acetic acid (5 drops) were added and the mixture was heated at reflux for 4h, then cooled down. The formed precipitate was filtered off and washed with water to give the title compound **19** (white solid, yield 0.42 g, 57%, mp 184–185 ° C (from propan-2-ol).

^1^H NMR (400 MHz, DMSO–*d_6_*) δ: 1.95 (s, 6H, 2CH_3_), 2.68–2.87 (m, 2H, CH_2_CO), 3,34–3.39 (m, 1H, CH), 3.97–4.16 (m, 2H, NCH_2_), 5.78 (s, 2H, CH-CH), 7.26 (d, *J =* 8.6 Hz, 2H, H_Ar_), 7.79 (d, *J =* 8.6 Hz, 2H, H_Ar_), 12.82 (s, OH) ppm.

^13^C NMR (101 MHz, DMSO–*d_6_*) δ: 12.86 (CH_3_), 35.16, 35.27 (CH, CH_2_CO), 49.89 (NCH_2_), 105.79, 119.72, 127.61, 128.25, 133.94, 138.39 (C_Ar_), 172.07, 174.18 (C=O) ppm.

IR (KBr): v_max_ 3288 (OH); 1673; 1654 (C=O); 1156; 1127; 1108; 1100 (C-N) cm^−1^.

Calcd. for C_17_H_18_N_2_O_3_, %: C 68.44; H 6.08; N 9.39. Found, %: C 68.12; H 5.80; N 9.13.

*General procedure for the preparation of hydrazones* **20** *and* **21**. To a hot solution of hydrazide **17** (0.59 g, 2.5 mmol) in water (60 mL) with the addition of hydrochloric acid (5 drops), the solution of the corresponding carbaldehyde (5.5 mmol) in propan-2-ol (5 mL) was added, and the mixture was heated at reflux for 2 (**20**) or 12 (**21**) h, then cooled down. The obtained solid was filtered off, washed with water, and dried to give the title compound **20** (white solid, yield 0.60 g, 57.3%, mp 268–269 °C (from propan-2-ol) or compound **21** (white solid, yield 0.86 g, 66.8%, mp 272–273 °C (from propan-2-ol).

*5-Oxo-N′-(thiophen-2-ylmethylene)-1-(4-((thiophen-2-ylmethylene)amino)phenyl)pyrrolidine-3-carbohydrazide* (**20**).

^1^H NMR (400 MHz, DMSO–*d_6_*) δ: (*Z/E*, 65/35) 2.68–2.85 (m, 2H, CH_2_CO), 3.27–3.35 (m, 0.35H, CH), 3.88–4.14 (m, 2H, NCH_2_, 0.65H, CH), 6.92 (d, *J* = 8.5 Hz, 1H, H_Ar_), 7.10–7.15 (m, 1H, H_Ar_), 7.15–7.41 (m, 2H, H_Ar_), 7.42–7.82 (m, 6H, H_Ar_), 8.21, 8.22, 8.31, 8.43, 8.44 (5s, 1H, N=CH), 8.78, 8.81, 8.82, 8.85 (4s, 1H, N=CH), 11.55, 11.57, 11.59, 11.61 (4s, 1H, NH) ppm.

^13^C NMR (101 MHz, DMSO–*d_6_*) δ: 32.99, 34.65, 34.80, 35.48 (CH_2_CO), 50.09, 50.33, 50.57, 50.80 (NCH_2_), 118.15, 118.16, 120.04, 120.08, 121.03, 121.07, 121.48, 127.85, 127.97, 128.25, 128.48, 128.99, 130.40, 131.01, 131.05, 133.43, 133.81, 137.45, 138.41, 138.64, 138.87, 138.96, 142.19, 142.60, 146.35, 153.05, 155.82 (C_Ar_), 168.52, 168.59, 171.37, 171.52, 171.81, 171.95, 173.11, 173.16 (C=O) ppm.

IR (KBr): v_max_ 3265 (NH); 1699; 1658 (C=O); 1514; 1509 (C=N); 1179 (C-N) cm^−1^.

Calcd. for C_21_H_18_N_4_O_2_S_2_, %: C 59.70; H 4.29; N 13.26. Found, %: C 59.80; H 4.39; N 12.96.

*N′-((5-nitrothiophen-2-yl)methylene)-1-(4-(((5-nitrothiophen-2-yl)methylene)amino)phenyl)-5-oxopyrrolidine-3-carbohydrazide* (**21**).

^1^H NMR (400 MHz, DMSO–*d_6_*) δ: (*Z/E*, 65/35) 2.72–2.91 (m, 2H, CH_2_CO), 3.34–3.41 (m, 0.35H, CH), 3.99–4.22 (m, 2H, NCH_2_, 0.65H, CH), 7.41–7.45 (m, 1H, H_Ar_), 7.51–7.58 (m, 1H, H_Ar_), 7.70 (d, *J* = 4.3 Hz, 1H, H_Ar_) 7.74–7.79 (m, 2H, H_Ar_), 8.07–8.13 (m, 1H, H_Ar_), 8.15–8.22 (m, 2H, H_Ar_), 8.48, 8.62, 8.79 (3s, 1H, N=CH), 8.93, 8.96, 9.01 (3s, 1H, N=CH), 11.99, 12.02 (2s, 1H, NH) ppm.

^13^C NMR (101 MHz, DMSO–*d_6_*) δ: 32.80, 34.85, 34.98, 35.60 (CH, CH_2_CO), 49.91, 50.32 (NCH_2_), 119.90, 119.93, 122.23, 129.05, 129.23, 129.78, 130.47, 130.61, 131.64, 134.42, 134.58, 136.13, 136.94, 138.65, 138.74, 140.62, 143.76, 144.68, 146.46, 146.56, 148.92, 150.55, 150.87, 152.17, 152.29, 156.49, 156.66, 157.32 (C_Ar_), 169.16, 171.91, 172.07, 173.77 (C=O) ppm.

IR (KBr): v_max_ 3265 (NH); 1699; 1657 (C=O); 1514; 1509 (C=N); 1179 (C-N) cm^−1^.

Calcd. for C_21_H_16_N_6_O_6_S_2_, %: C 49.21; H 3.15; N 16.40. Found, %: C 49.25; H 3.25; N 16.20.

*N-(2,5-dimethyl-1H-pyrrol-1-yl)-1-(4-(2,5-dimethyl-1H-pyrrol-1-yl)phenyl)-5-oxopyrrolidine-3-carboxamide* (**22**). To a solution of compound **17** (0.5 g, 2.1 mmol) in methanol (20 mL) hexane-2,5-dione (0.96 g, 8.4 mmol) and glacial acetic acid (5 drops) were added and the mixture was heated at reflux for 4 h, then cooled down. The formed precipitate was filtered off and washed with water to give the title compound **22** (white solid, yield 0.26 g, 32%, mp 245–246 °C (from propan-2-ol).

^1^H NMR (400 MHz, DMSO–*d_6_*) δ: 1.96 (s, 6H, 2CH_3_), 2.01 (s, 6H, 2CH_3_), 2.71–3.01 (m, 2H, CH_2_CO), 3.44–3.54 (m, 1H, CH), 3.98–4.26 (m, 2H, NCH_2_), 5.65 (s, 2H, CH-CH), 5.79 (s, 2H, CH-CH), 7.28 (d, *J =* 8.3 Hz, 2H, H_Ar_), 7.82 (d, *J =* 8.3 Hz, 2H, H_Ar_), 10.94 (s, 1H, NH) ppm.

^13^C NMR (101 MHz, DMSO–*d_6_*) δ: 10.95, 10.98, 12.87 (CH_3_), 34.04, 35.74, 50.30 (CH_2_CO, CH, NCH_2_), 103.10, 105.80, 119.77, 126.74, 127.61, 128.28, 133.99, 138.34 (C_Ar_), 171.85, 171.88 (C=O) ppm.

IR (KBr): v_max_ 3265 (NH), 1699; 1658 (C=O); 1514 (C=N); 1235; 1203; 1179; 1145; 1128 (C-N) cm^−1^.

Calcd. for C_23_H_26_N_4_O_2_, %: C 70.75; H 6.71; N 14.35. Found, %: C 70.58; H 6.64; N 14.18.

### 3.2. Antimicrobial Activity Characterization

#### 3.2.1. Bacterial Strains and Culture Conditions

The multidrug-resistant and genetically defined isolates were obtained from the ARisolate bank at the Centre for Disease Control (CDC, Atlanta, Georgia, USA). *S. aureus* TCH 1516 (USA300), *B. fragilis*, and *P. gingivalis* were obtained from American Type Culture Collection. Prior to the study, all strains were maintained in commercial cryopreservation systems at −80 °C. Aerobic bacterial strains were subcultured on Columbia Sheep Blood agar (Becton Dickenson, Franklin Lakes, NJ, USA). Anaerobic bacteria were cultured on Anaerobic Blood agar in sealed commercial cultivation chambers (GasPak, Franklin Lakes, NJ, USA). Unless otherwise specified, all antimicrobial susceptibility studies were performed in Cation-Adjusted Mueller–Hinton broth (CAMBH) for liquid cultures (Liofilchem, Via Scozia, Italy).

#### 3.2.2. Minimal Inhibitory Concentration Determination

The minimal inhibitory concentrations (MICs) of compounds **2** and **4**–**22,** as well as various antibiotics, were determined according to the recommendations of the Clinical and Laboratory Standards Institute (CLSI) [38]. The MICs for the compounds and comparator antibiotics were determined according to the testing standard broth microdilution methods described in CLSI document M07-A8 against the libraries of Gram-positive and Gram-negative pathogens. The compounds and antibiotics were dissolved in dimethyl sulfoxide (DMSO) to achieve a final concentration of 30 mg/mL. Series of dilutions were prepared in deep 96-well microplates to achieve 2× of assay concentrations (0.5–64 µg/mL) and were then transferred to the assay plates. A standardized inoculum was prepared using direct colony suspension. Within 15 min of preparation, the adjusted inoculum suspension was diluted in sterile CAMBH to achieve final concentrations of approximately 5 × 10^5^ CFU/mL (range, 2 × 10^5^ to 8 × 10^5^ CFU/mL) in each well. The inoculum was transferred to the assay plates to achieve a 1× assay concentration. For the anaerobic pathogens, CAMBH was replaced with CAMBH supplemented with vitamin K, leaked horse blood and plates were incubated in an anaerobic environment. Inoculated microdilution plates were incubated at 35 °C for 16 to 20 h in an ambient-air incubator within 15 min of the addition of the inoculum.

#### 3.2.3. The Cytotoxic Activity Characterization

The A549 and HSAEC1-KT cells were obtained from American Type Culture Collection. The cytotoxic activity of compounds **2** and **4**–**22,** as well as cisplatin (Sigma-Aldrich, Saint Louis, Missouri, USA), was determined by using an MTT assay (ThermoFisher Scientific, Waltham, Massachusetts USA). Briefly, cells were plated in 96-well plates at a density of 1 × 10^4^ cells/well in DMEM with 10% FBS (for A549) or SAGM BulletKit medium (Lonza CC-3119 and CC-4124) containing supplements (AGM™ SingleQuots™, Lonza CC-4124) (for HSAEC1-KT). After overnight attachment at 37 °C, 5% CO_2_, cells were treated with compounds (100 µM) in triplicate. After 20 h treatment, the MTT reagent was added, and cells were further incubated for 4 h. The formazan was then extracted with anhydrous DMSO. The samples were measured using a microplate reader at a wavelength of 570 nm. The following formula was used to calculate the percentage of A549 viability: ([AE-AB]/[AC-AB]) × 100%. AE, AC, and AB were defined as the absorbance of experimental samples, untreated samples, and blank controls, respectively.

#### 3.2.4. Statistical Analysis

The data are expressed as a mean ± SD value from three separate experiments unless stated otherwise. The statistical significance was determined using a test. Data were considered significant when *p* < 0.05.

## 4. Conclusions

In conclusion, the synthesis, chemical transformations, and biological assessment of some 1-(4-acetamidophenyl)- and 1-(4-aminophenyl)-5-oxopyrrolidine derivatives bearing hydrazone and azole fragments are provided herein. All the prepared compounds were characterized using spectroscopic techniques and elemental analysis.

Compound **21** showed promising and selective antimicrobial activity targeting multidrug-resistant *Staphylococcus aureus* harboring emerging multidrug-resistance mechanisms. The activity of compound **21** was comparable to or greater than clinically approved antibiotics against *S. aureus* with challenging resistance mechanisms, demonstrating the great potency of compound **21** for the further hit to lead optimization. It is worth mentioning that the in vitro antimicrobial activity exhibited by compound **21** is reduced in linezolid/tedizolid-resistant *S. aureus* strains, suggesting that pre-existing resistance mechanisms conferring *S. aureus* resistance to linezolid/tedizolid perhaps can overcome compound **21** mediated antimicrobial activity.

In addition to that, compounds **18**–**21** showed promising anticancer activity against A549 human lung adenocarcinoma cells, demonstrating that the 1-(4-aminophenyl)-5-oxopyrrolidine scaffold could be further explored as a promising anticancer pharmacophore for the development and optimization of novel antimicrobial and anticancer candidates.

## Figures and Tables

**Table 1:** The antimicrobial activity of 5-oxopyrrolidine derivative **21** against multidrug-resistant and vancomycin intermediate resistant *Staphylococcus aureus* strains. The data expressed in the table represents minimal inhibitory concentration from 3 technical replicates.

Bacteria	Strain Number	Resistance Mechanisms	MIC, µg/mL				
			Compound 21	FOX	CLIN	VAN	LZD
*S. aureus*	215	*aadD, blaZ, erm(A), mecA, spc*	8	>16	32	4	4
*S. aureus*	216	*aph(3′)-III, mecA, mph(C), msr(A)*	2	16	0.25	4	2
*S. aureus*	219	*aac(6′)-aph(2″), aadD, erm(A), mecA, spc, tet(M)*	4	>16	>16	8	1
*S. aureus*	223	*mecA*	1	16	0.5	2	4
*S. aureus*	224	*aph(3′)-III, erm(A), mecA, spc, tet(K)*	4	16	1	4	4
*S. aureus*	227	*aadD, blaZ, erm(A), mecA, spc*	8	>16	16	4	4

**Abbreviations:** FOX—cefoxitin, CLIN—clindamycin, VAN—vancomycin, LZD—linezolid.

**Table 2:** The antimicrobial activity of 5-oxopyrrolidine derivative **21** against linezolid/tedizolid-resistant *Staphylococcus aureus* strains. The data expressed in the table represents minimal inhibitory concentration from 3 technical replicates.

Bacteria	Strain Number	Resistance Mechanisms	MIC, µg/mL					
			Compound 21	FOX	CLIN	VAN	LZD	TED
*S. aureus*	701	*mecA*	16	16	1	1	16	1
*S. aureus*	702	*mecA*	4	16	0.5	1	8	1
*S. aureus*	703	*mecA*	32	16	32	0.5–1	8	2
*S. aureus*	704	*mecA*	64	16	1	1	32	2

**Abbreviations:** FOX—cefoxitin, CLIN—clindamycin, VAN—vancomycin, LZD—linezolid, TED—tedizolid.

**Table 3:** The antimicrobial activity of 5-oxopyrrolidine derivative **21** against clinically significant Gram-positive and Gram-negative anaerobic bacteria strains. The data expressed in the table represents minimal inhibitory concentration from 3 technical replicates.

Bacterial Strains	MIC, µg/mL	
	Compound 21	Metronidazole
*Clostridioides difficile* AR-1074	16	1
*Clostridium perfringens* ATCC 12916	8	0.5
*Bacteroides fragilis* ATCC 43858	64	0.5
*Porphyromonas gingivalis* ATCC 53978	32	0.5

## Data Availability

Data is contained within the article and Supplementary Materials. The compounds are available from the corresponding author.

## Supplementary Materials

The following supporting information can be downloaded at: https://www.mdpi.com/article/10.3390/ph15080970/s1, Figure S1: ^1^H NMR of compound **2**; Figure S2: ^13^C NMR of compound **2**; Figure S3: ^1^H NMR of compound **4**; Figure S4: ^13^C NMR of compound **4**; Figure S5: ^1^H NMR of compound **5**; Figure S6: ^1^H NMR of compound **6**; Figure S7: ^13^C NMR of compound **6**; Figure S8: ^1^H NMR of compound **7**; Figure S9: ^1^H NMR of compound **8**; Figure S10: ^1^H NMR of compound **9**; Figure S11: ^1^H NMR of compound **10**; Figure S12: ^1^H NMR of compound **11**; Figure S13: ^1^H NMR of compound **12**; Figure S14: ^1^H NMR of compound **13**; Figure S15: ^1^H NMR of compound **14**; Figure S16: ^13^C NMR of compound **14**; Figure S17: ^1^H NMR of compound **15**; Figure S18: ^13^C NMR of compound **15**; Figure S19: ^1^H NMR of compound **16**; Figure S20: ^13^C NMR of compound **16**; Figure S21: ^1^H NMR of compound **17**; Figure S22: ^13^C NMR of compound **17**; Figure S23: ^1^H NMR of compound **18**; Figure S24: ^13^C NMR of compound **18**; Figure S25: ^1^H NMR of compound **19**; Figure S26: ^13^C NMR of compound **19**; Figure S27: ^1^H NMR of compound **20**; Figure S28: ^13^C NMR of compound **20**; Figure S29: ^1^H NMR of compound **21**; Figure S30: ^13^C NMR of compound **21**; Figure S31: ^1^H NMR of compound **22**; Figure S32: ^13^C NMR of compound **22**; Table S1. The antimicrobial activity of compounds **2**, **4**–**22**.
