# Supplementary material for: Synthesis and Biological Activity Characterization of Novel 5-Oxopyrrolidine Derivatives with Promising Anticancer and Antimicrobial Activity
Source: Pharmaceuticals (Basel). 2022 Aug 6;15(8):970. doi: 10.3390/ph15080970 (PMC9415606; doi:10.3390/ph15080970)
Supplement: Supplementary file 1 [file pharmaceuticals-15-00970-s001.zip › pharmaceuticals-1825710-supplementary.pdf]

Supplementary Materials

Synthesis and biological activity characterization of novel 5-oxopyrrolidine derivatives with promising anticancer and antimicrobial activity

NMR Spectra (compounds 2, 4–22, all in DMSO-d_6, Figures S1–S32)
Supplementary Table S1.

1-(4-Acetamidophenyl)-5-oxopyrrolidin-3-carboxylic acid **(2)**

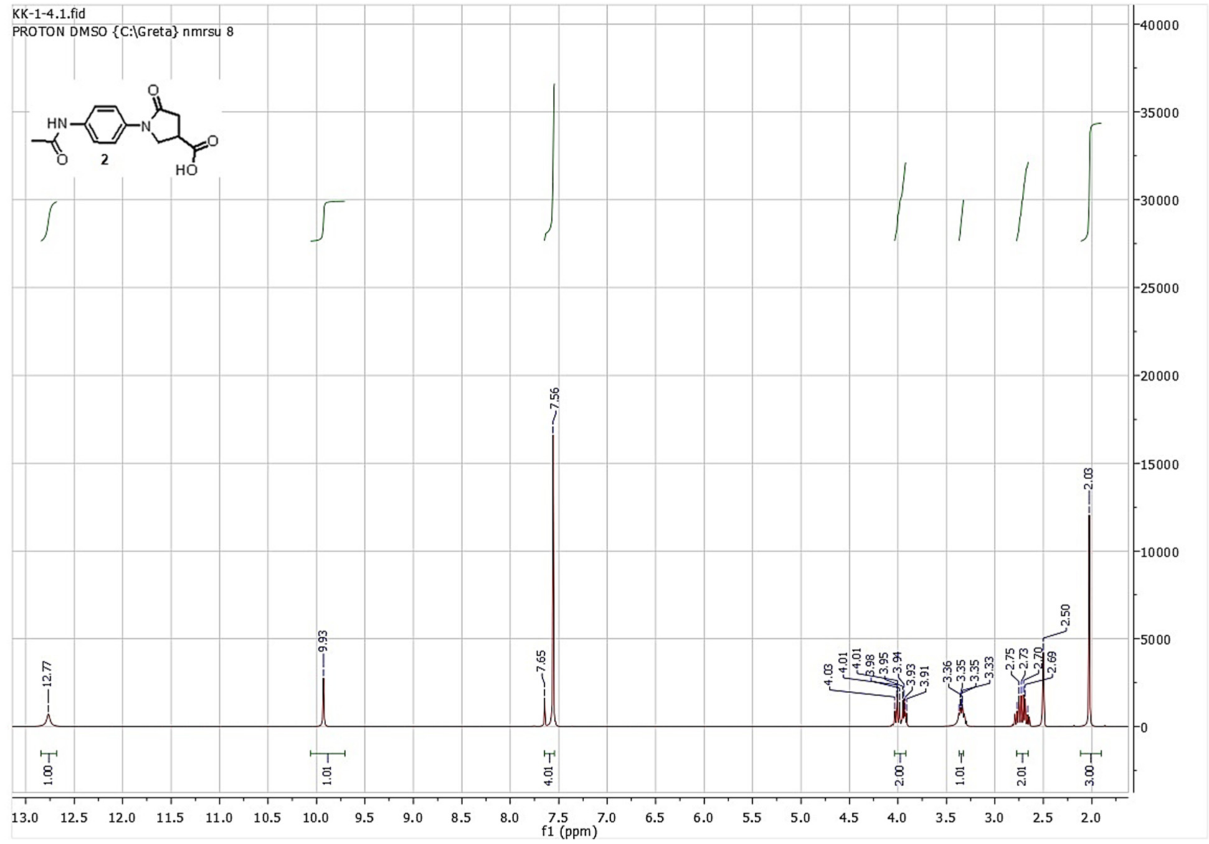

Figure S1. ^{1}H NMR of compound **2.**

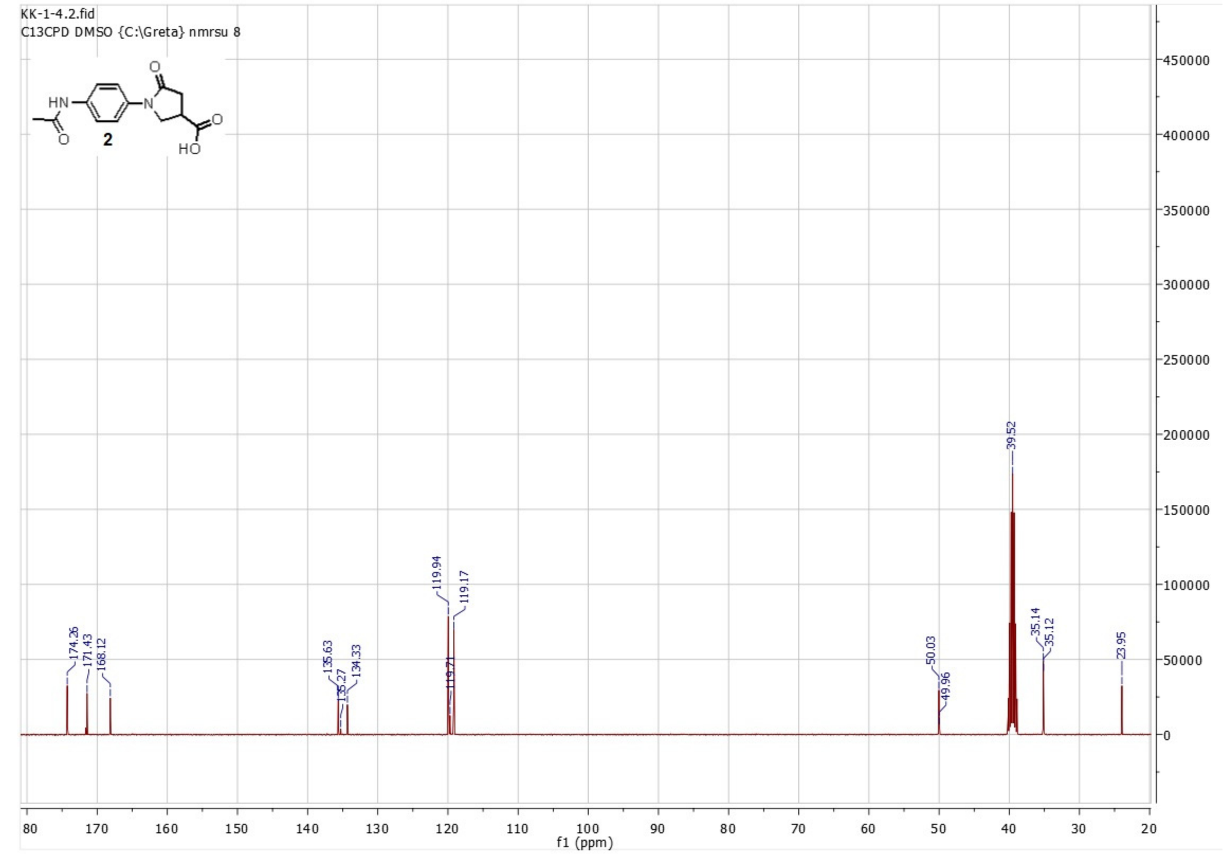

Figure S2. ^{13}C NMR of compound **2.**

N-(4-(4-(hydrazinecarbonyl)-2-oxopyrrolidin-1-yl)phenyl)acetamide **(4)**

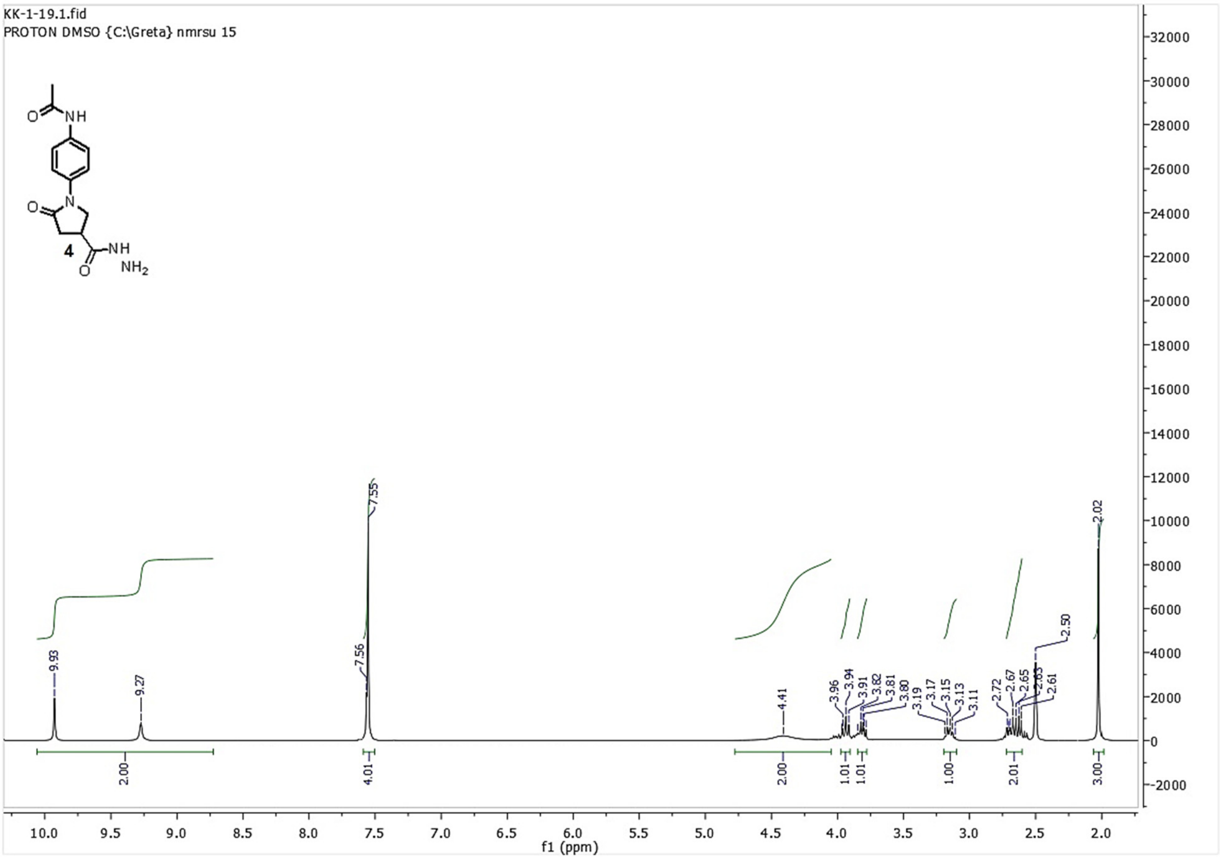

Figure S3. ^{1}H NMR of compound **4.**

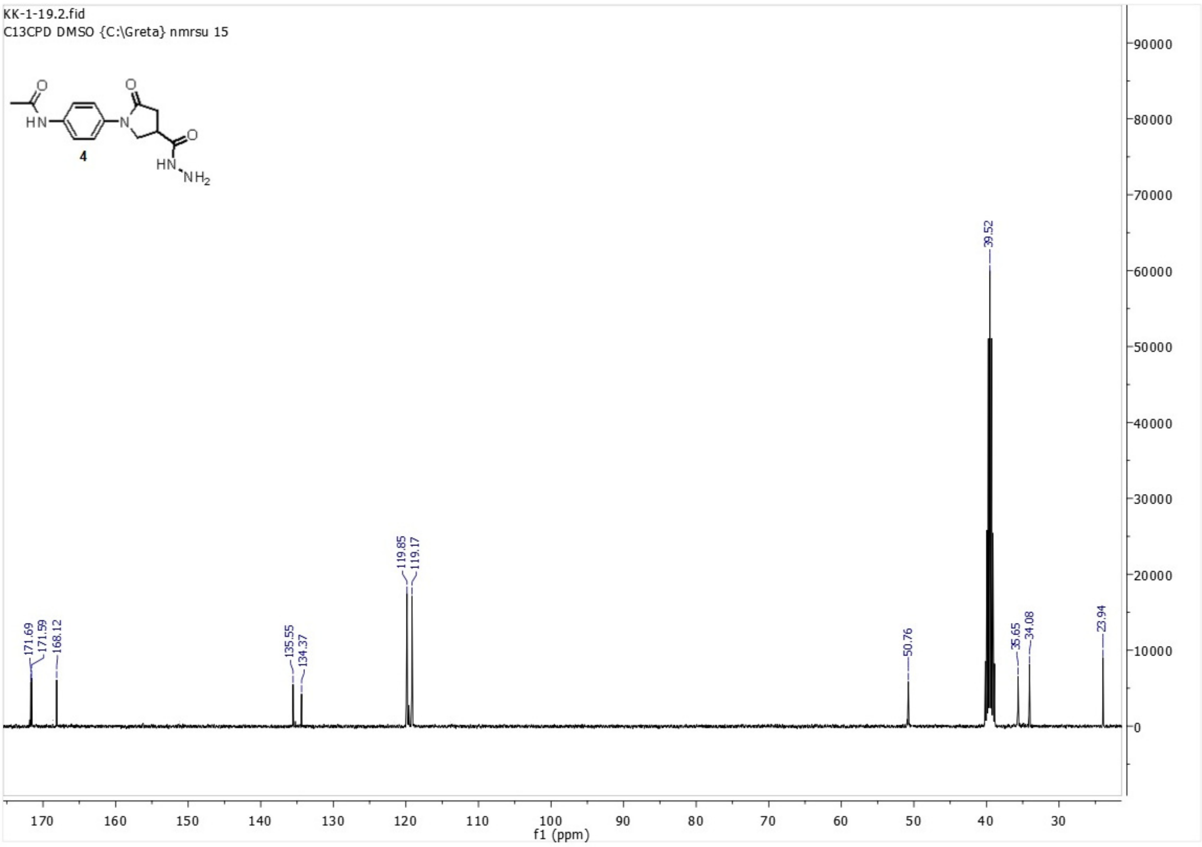

Figure S4. ^{13}C NMR of compound **4.**

N-(4-(4-(2-benzylidenehydrazine-1-carbonyl)-2-oxopyrrolidin-1-yl)phenyl)acetamide **(5)**.

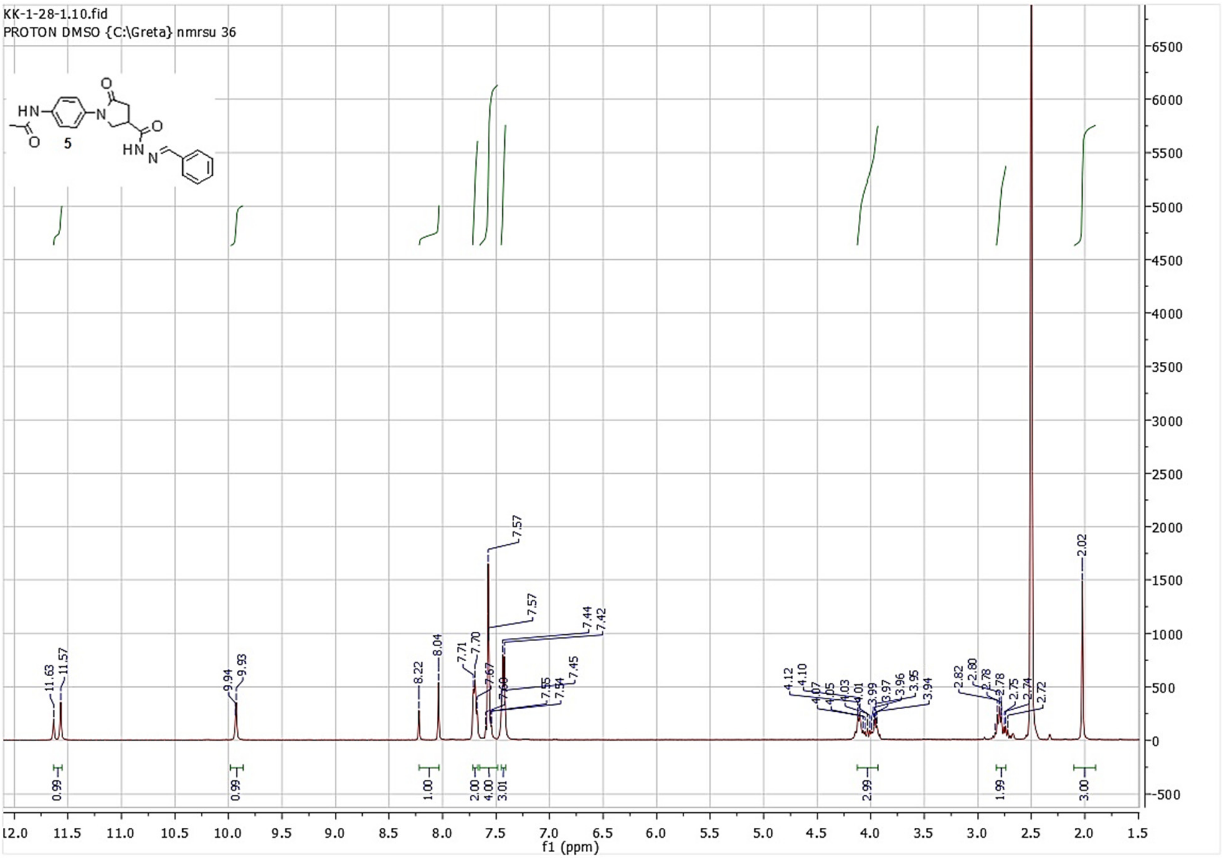

Figure S5. ^{1}H NMR of compound **5.**

N-(4-(4-(2-(4-chlorobenzylidene)hydrazine-1-carbonyl)-2-oxopyrrolidin-1-yl)phenyl)acetamide **(6)**.

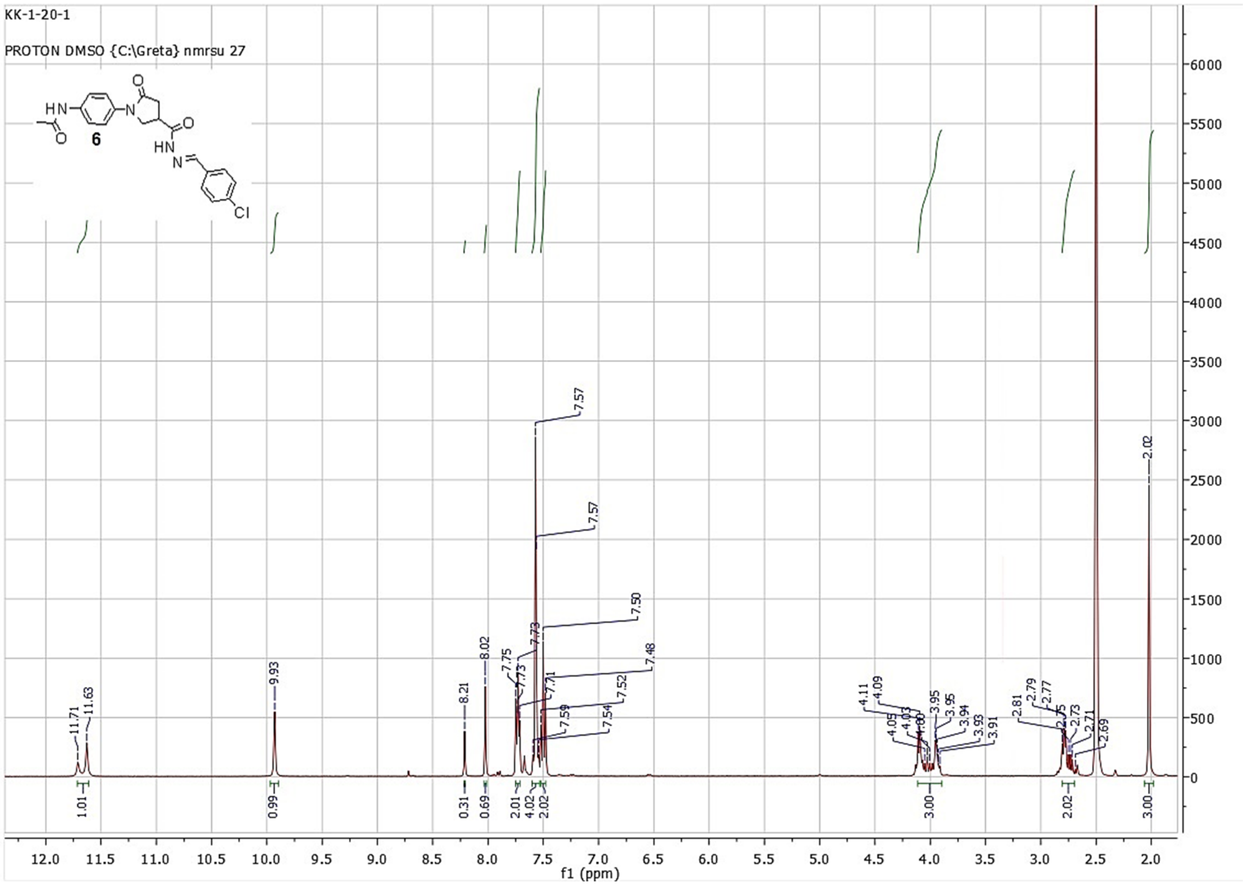

Figure S6. ^{1}H NMR of compound **6.**

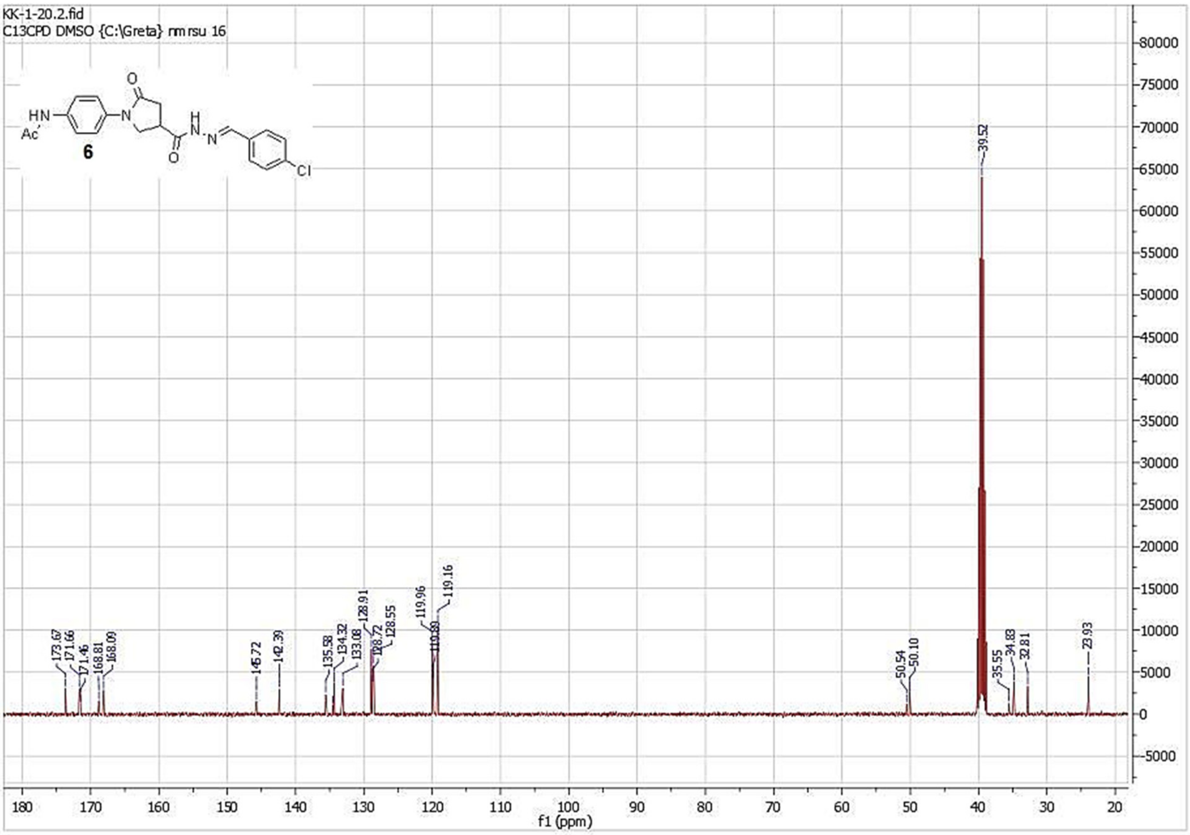

Figure S7. ^{13}C NMR of compound **6.**

N-(4-(4-(2-(4-bromobenzylidene)hydrazine-1-carbonyl)-2-oxopyrrolidin-1-yl)phenyl)acetamide **(7).**

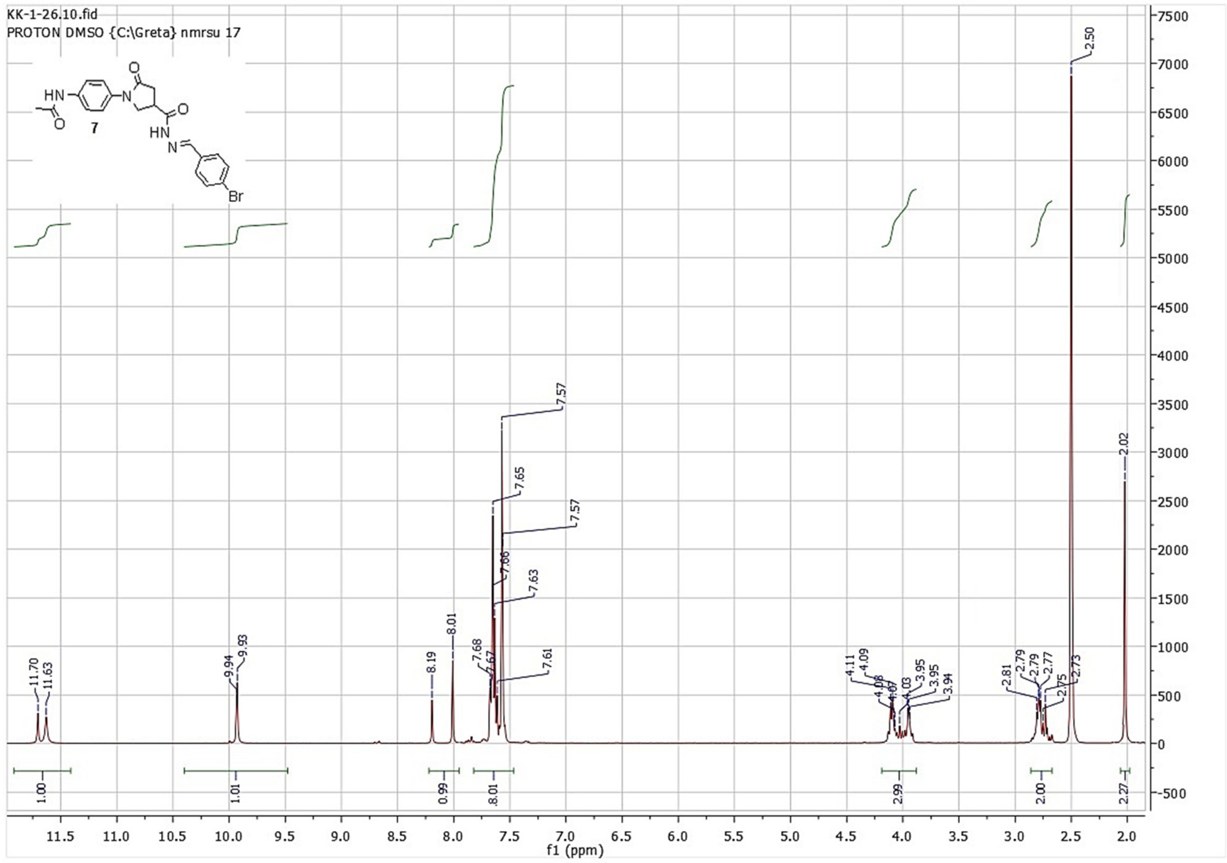

Figure S8. ^{1}H NMR of compound **7.**

N-(4-(4-(2-(4-(dimethylamino)benzylidene)hydrazine-1-carbonyl)-2-oxopyrrolidin-1-yl)phenyl)acetamide **(8)**.

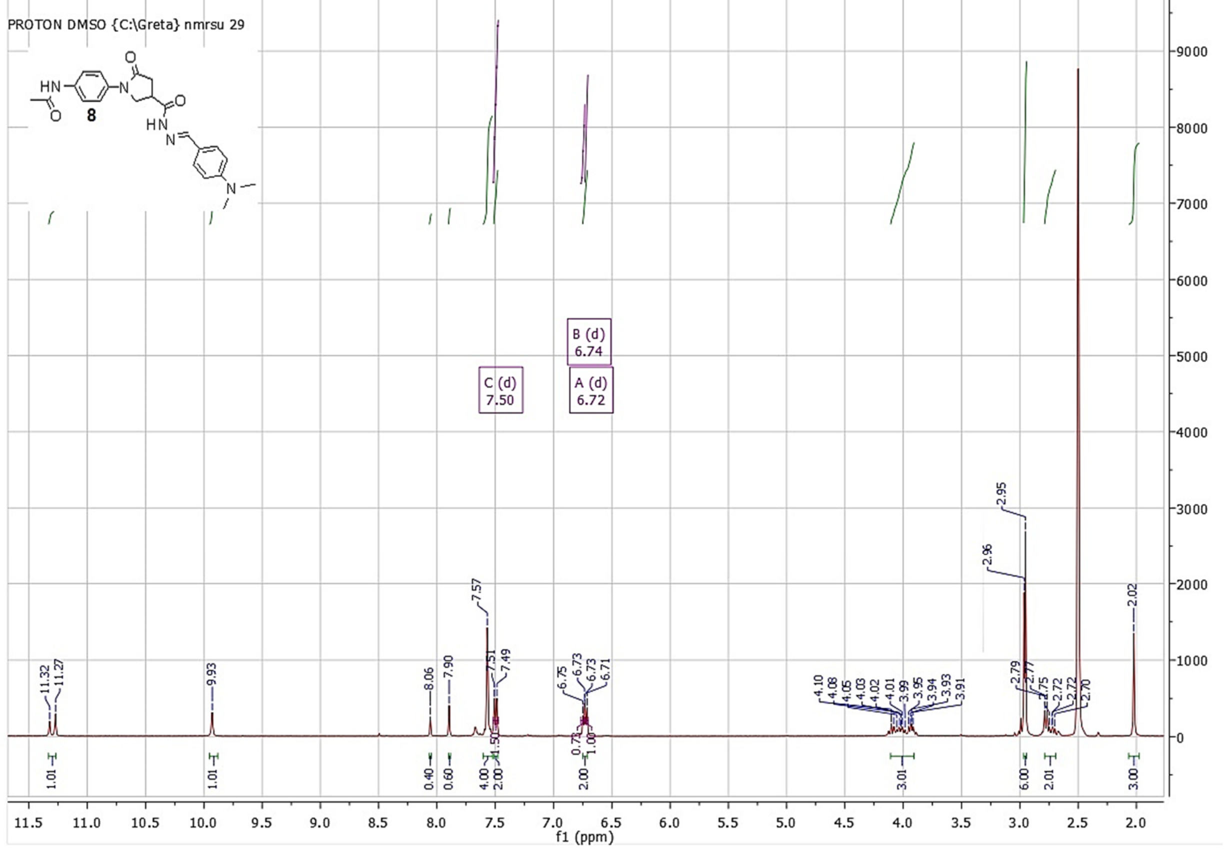

Figure S9. ^{1}H NMR of compound **8.**

N-(4-(4-(2-(4-methoxybenzylidene)hydrazine-1-carbonyl)-2-oxopyrrolidin-1-yl)phenyl)acetamide **(9).**

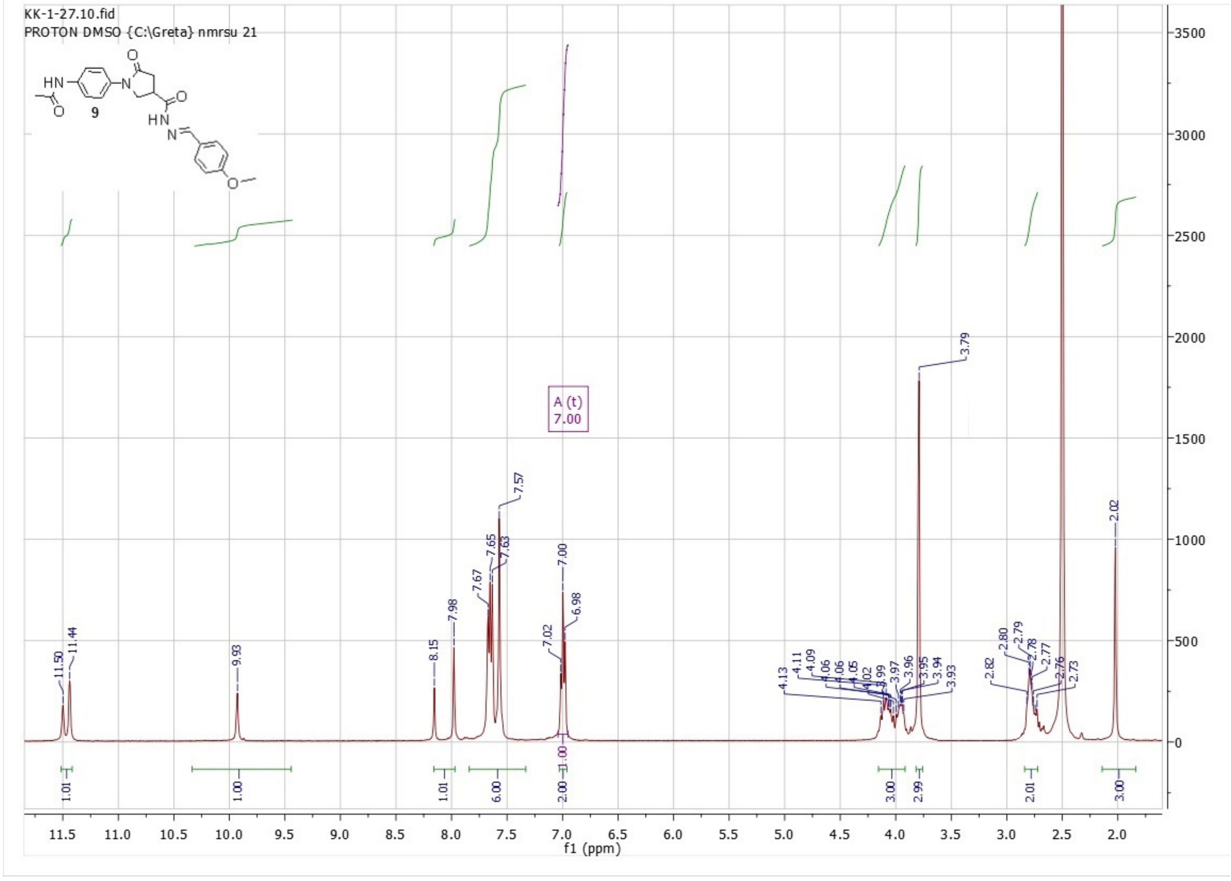

Figure S10. ^{1}H NMR of compound **9.**

N-(4-(4-(2-(2,5-dimethoxybenzylidene)hydrazine-1-carbonyl)-2-oxopyrrolidin-1-yl)phenyl)acetamide **(10).**

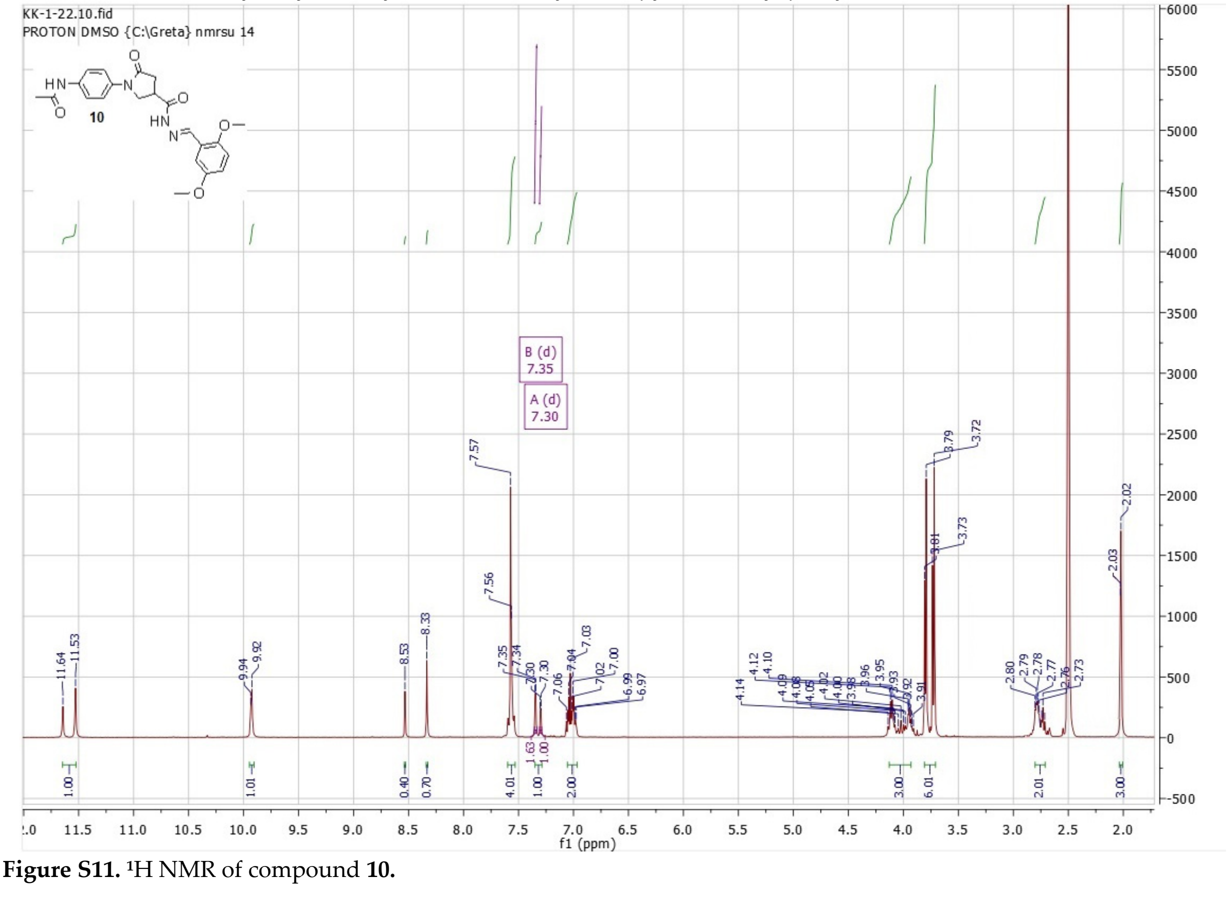

Figure S11. ^{1}H NMR of compound **10.**

N-(4-(2-oxo-4-(2-(2,4,6-trimethoxybenzylidene)hydrazine-1-carbonyl)pyrrolidin-1-yl)phenyl)acetamide **(11).**

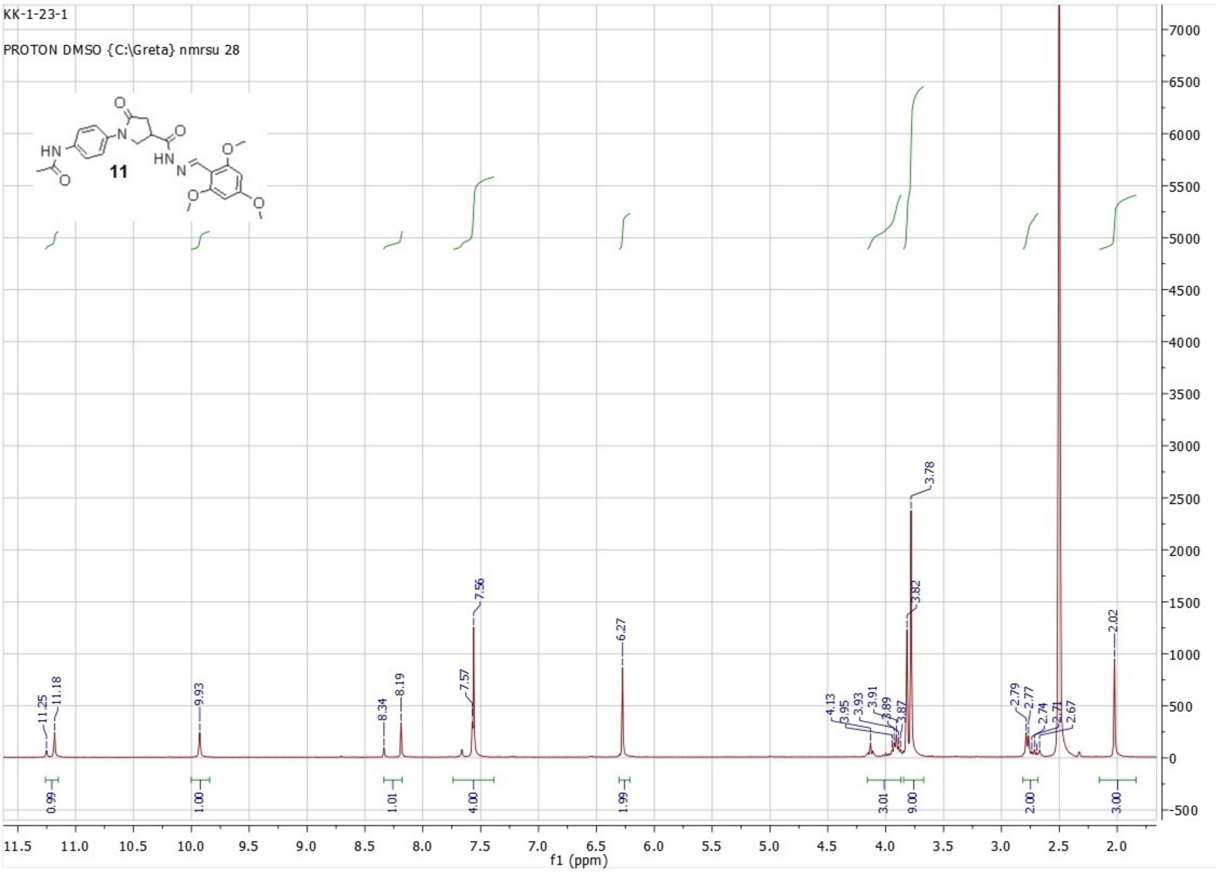

Figure S12. ¹H NMR of compound **11.**

N-(4-(2-oxo-4-(2-(propan-2-ylidene)hydrazine-1-carbonyl)pyrrolidin-1-yl)phenyl)acetamide **(12).**

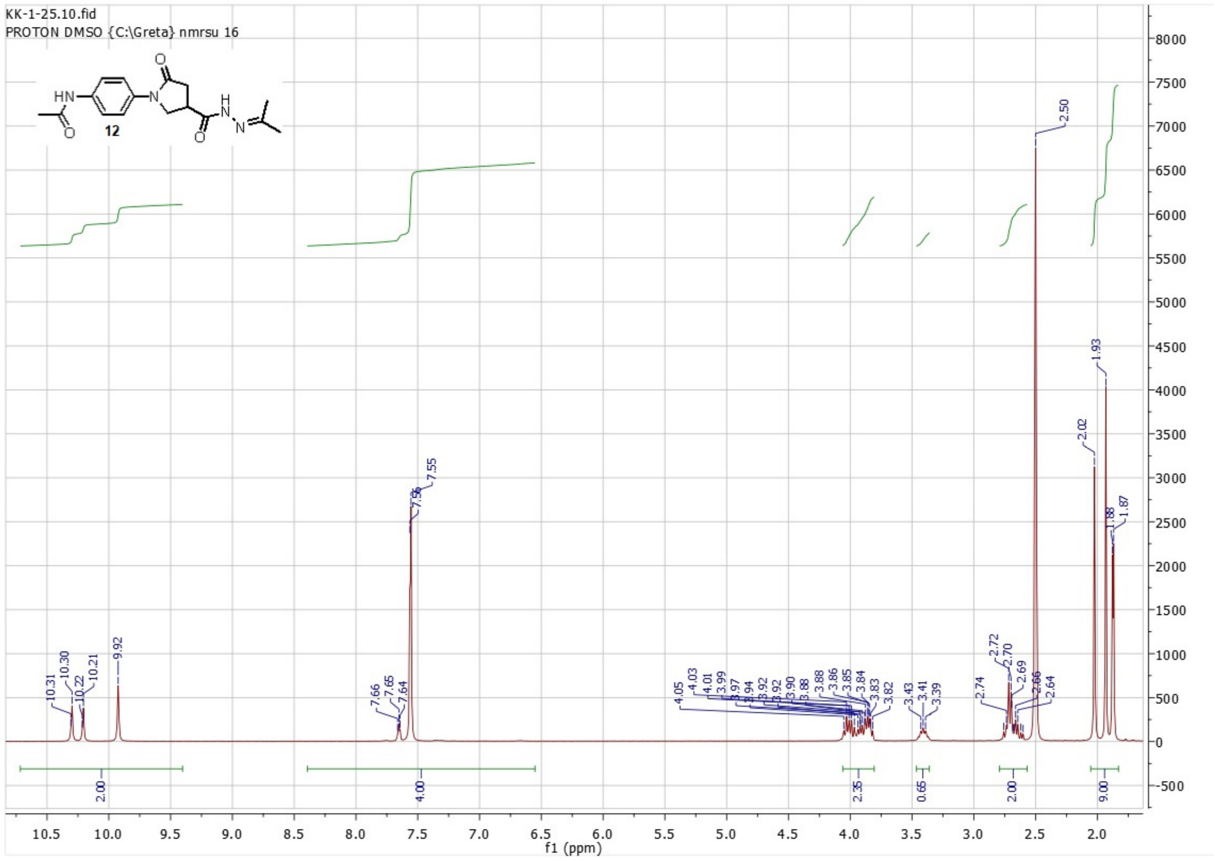

Figure S13. ¹H NMR of compound **12.**

N-(4-(4-(2-(butan-2-ylidene)hydrazine-1-carbonyl)-2-oxopyrrolidin-1-yl)phenyl)acetamide **(13)**.

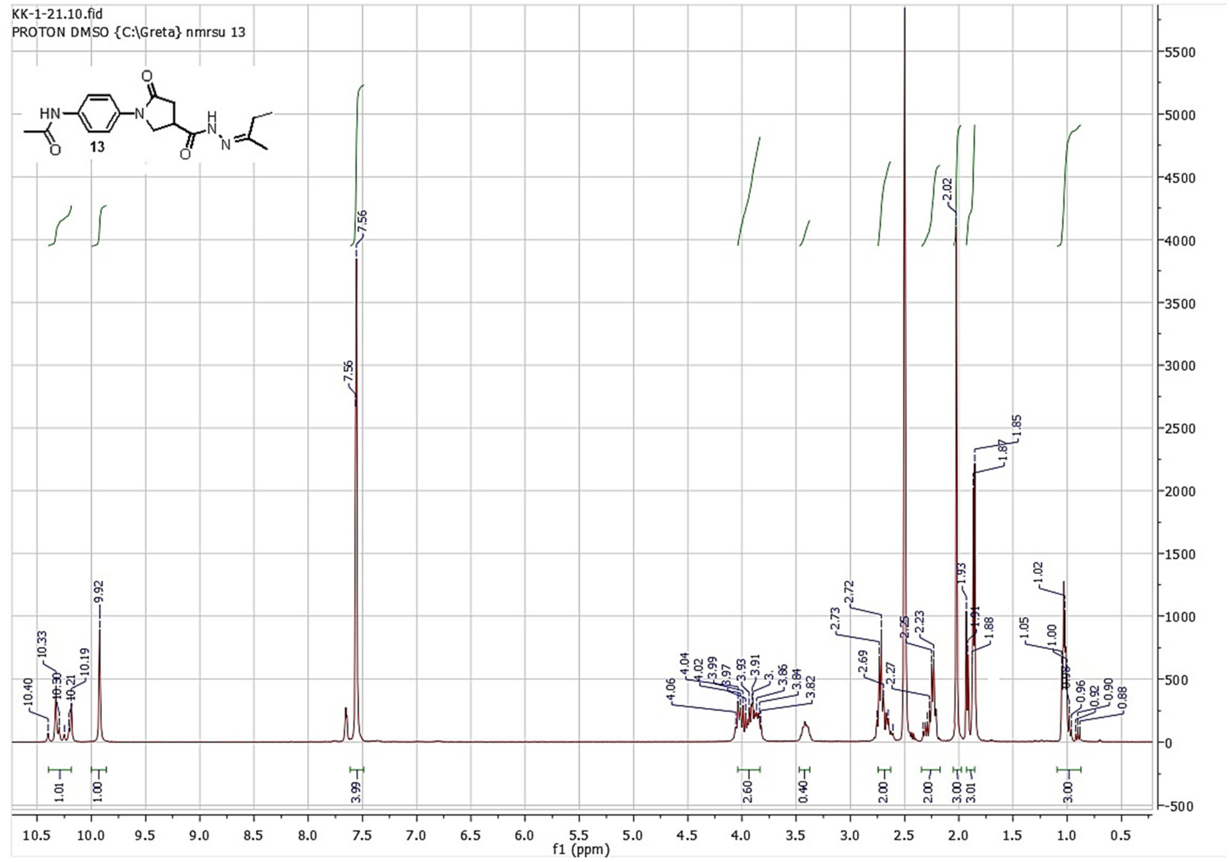

Figure S14. ¹H NMR of compound **13.**

N-(4-(4-(3,5-dimethyl-1H-pyrazol-1-carbonyl)-2-oxopyrrolidin-1-yl)phenyl)acetamide **(14)**.

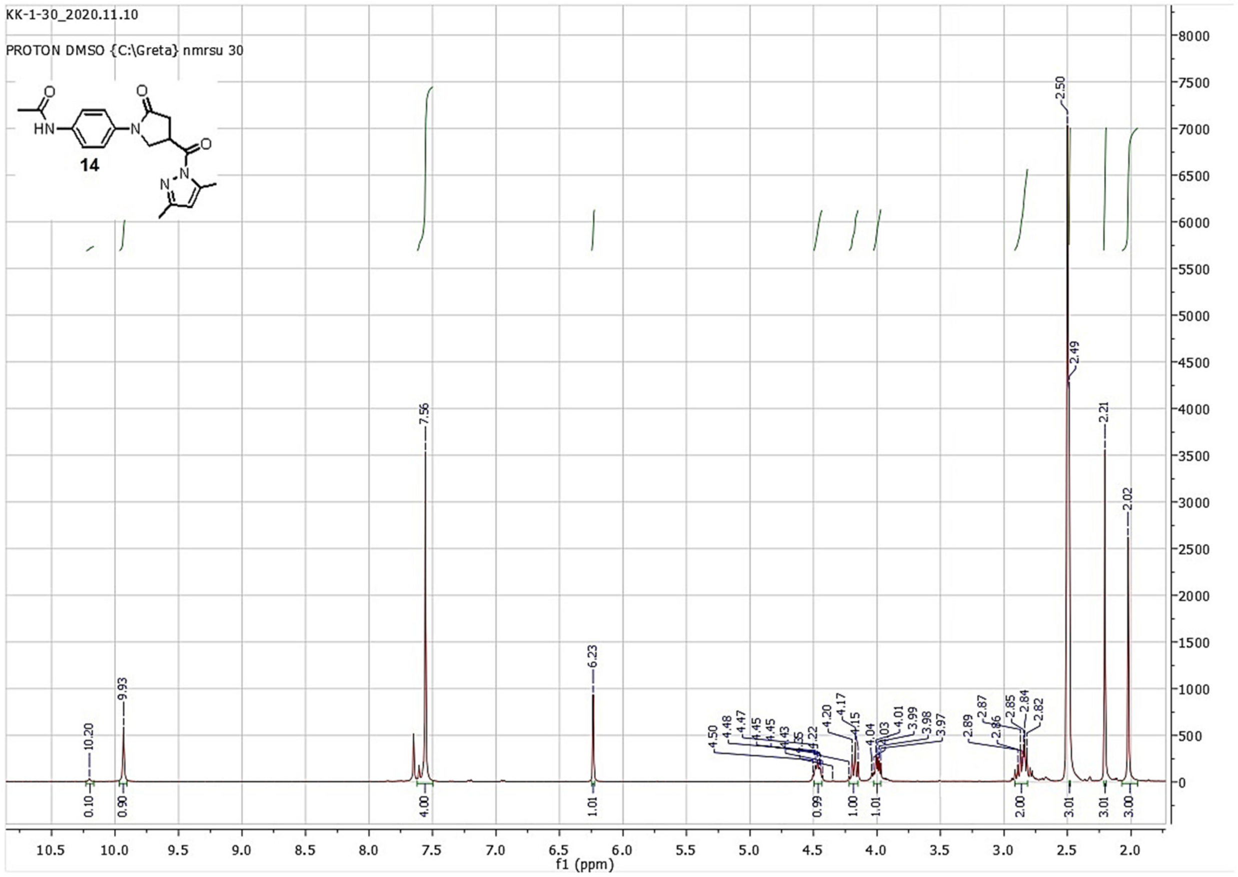

Figure S15. ^{1}H NMR of compound **14.**

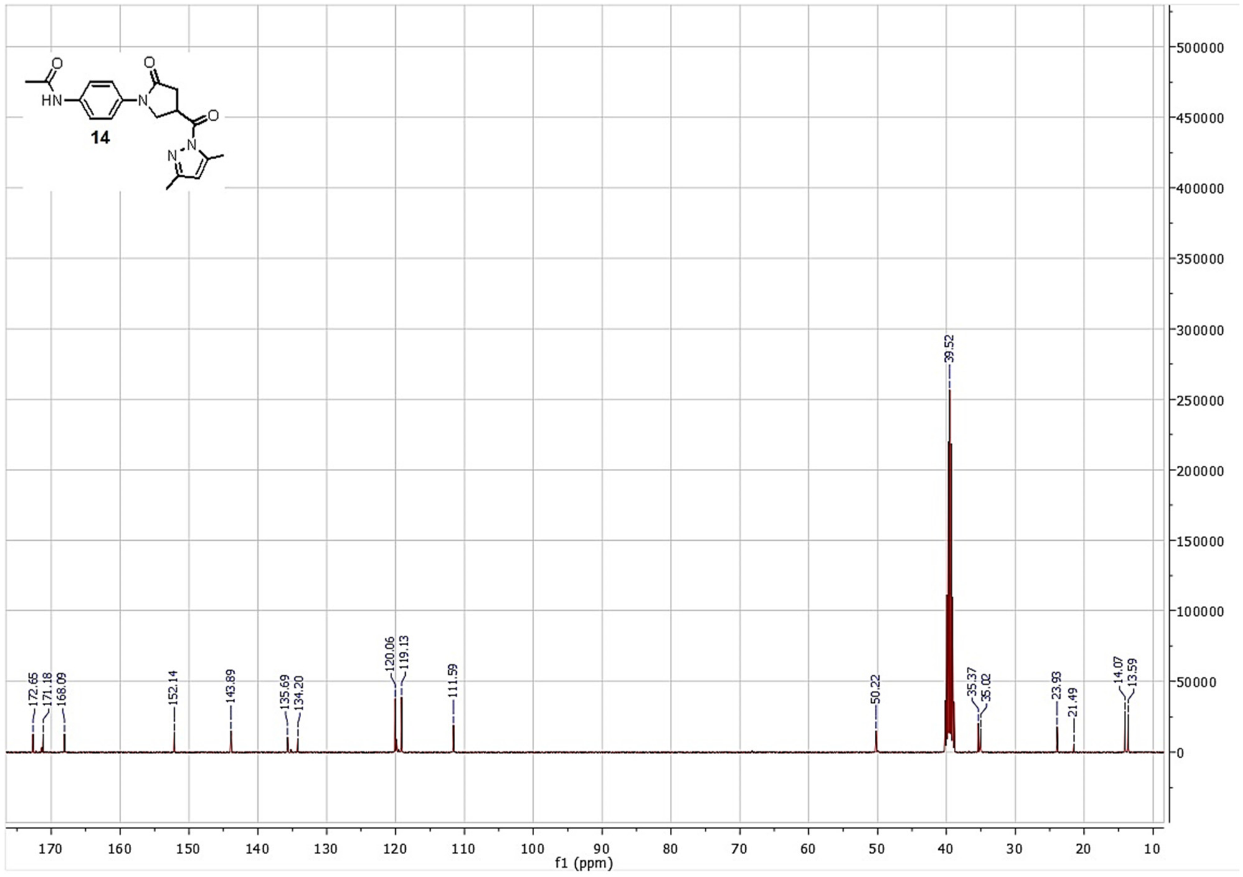

Figure S16. ^{13}C NMR of compound **14.**

N-(4-(4-(2-(2,5-dimethyl-1H-pyrrol-1-yl)acetyl)-2-oxopyrrolidin-1-yl)phenyl)acetamide **(15)**.

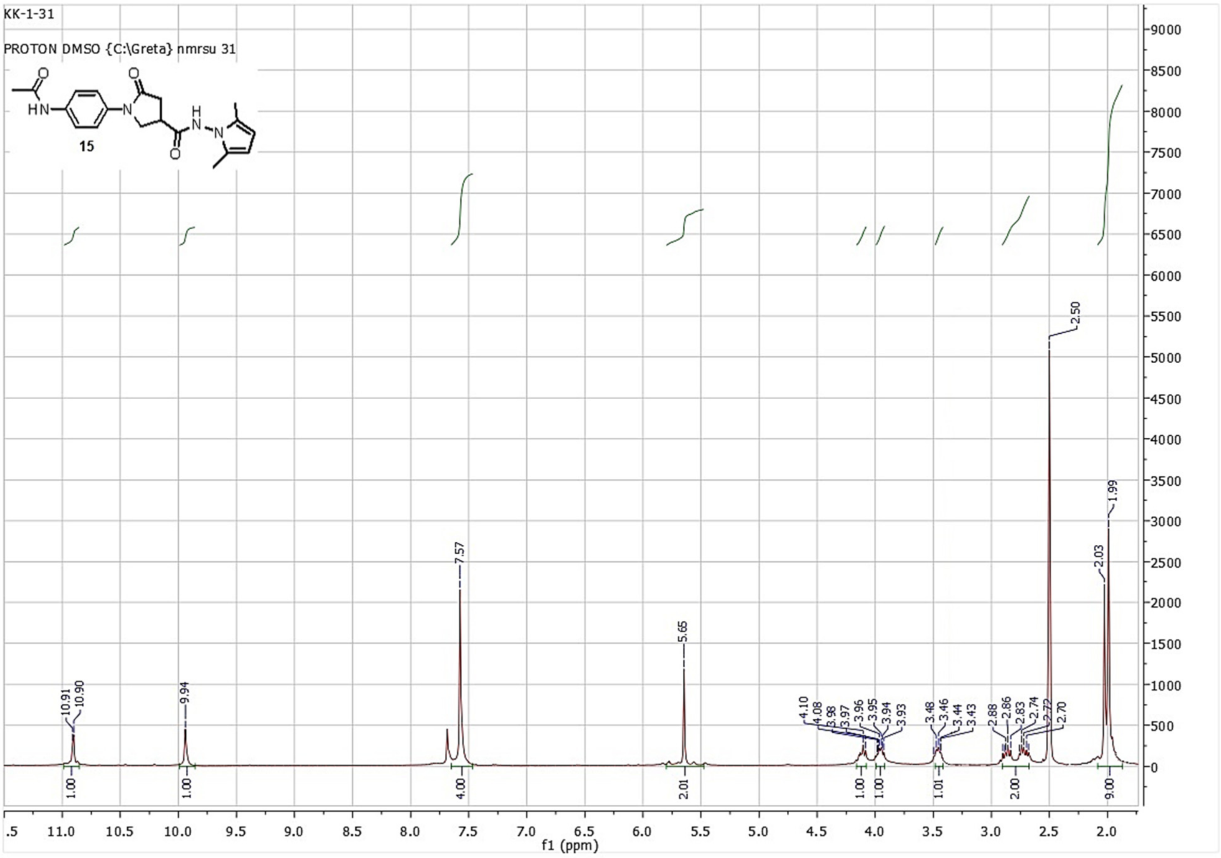

Figure S17. ¹H NMR of compound **15.**

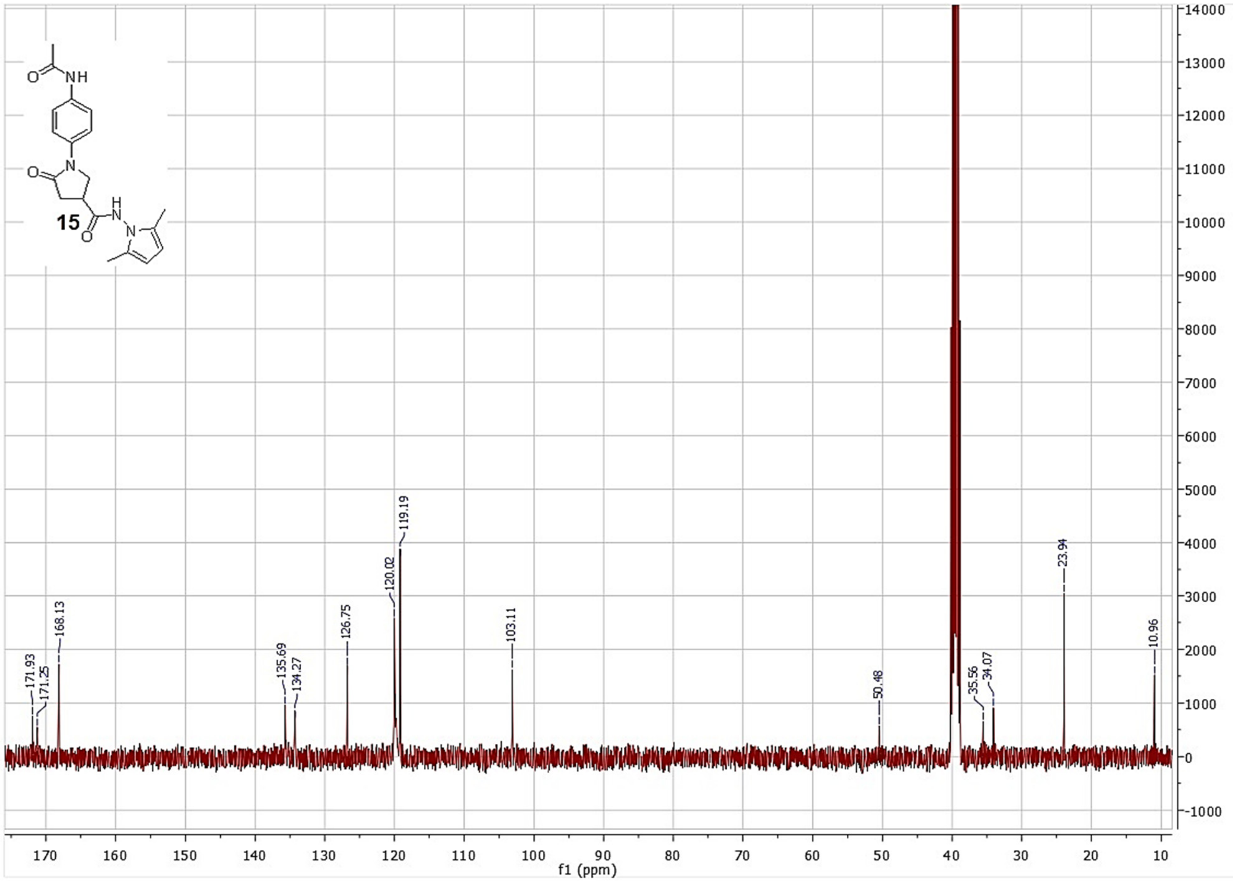

Figure S18. ¹³C NMR of compound **15.**

1-(4-Aminophenyl)-5-oxopyrrolidine-3-carboxylic acid **(16)**.

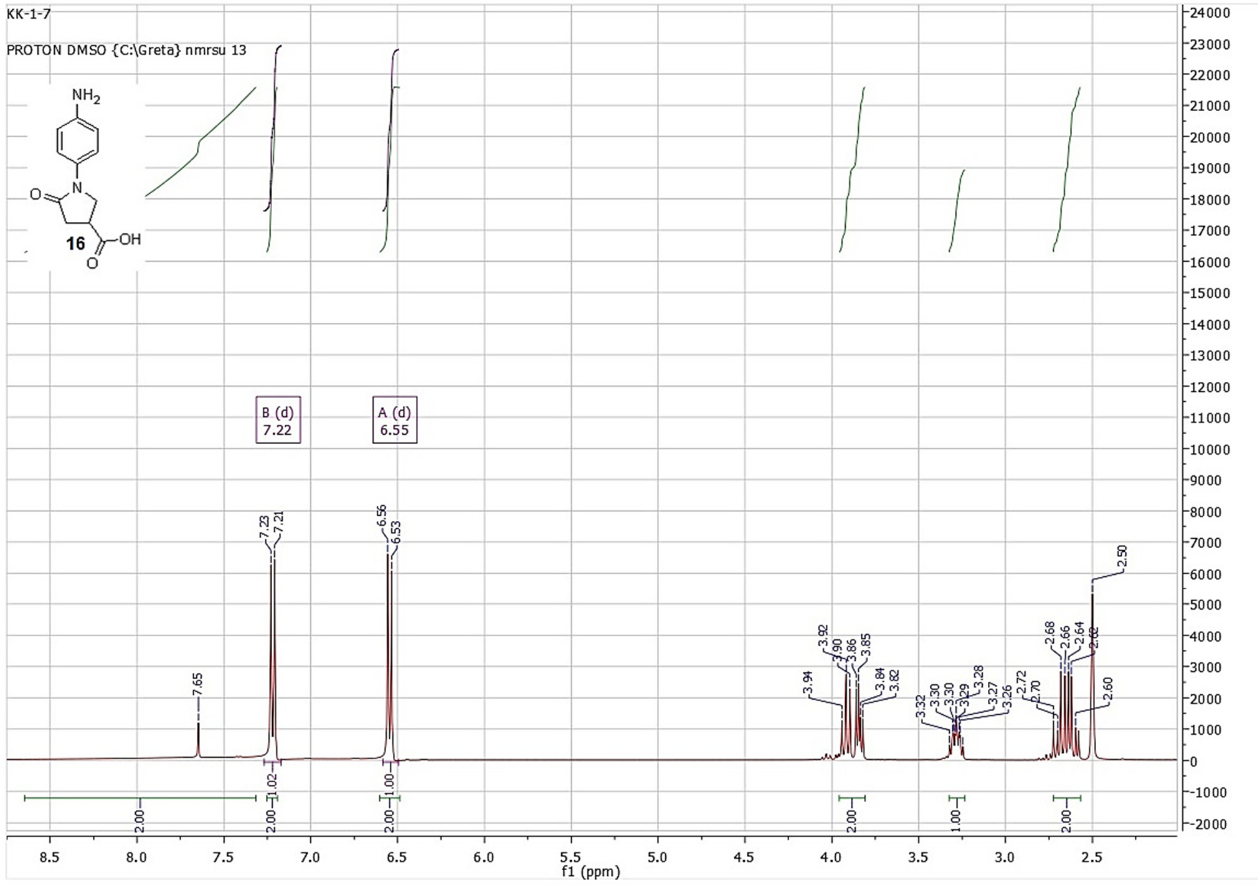

Figure S19. ^{1}H NMR of compound **16.**

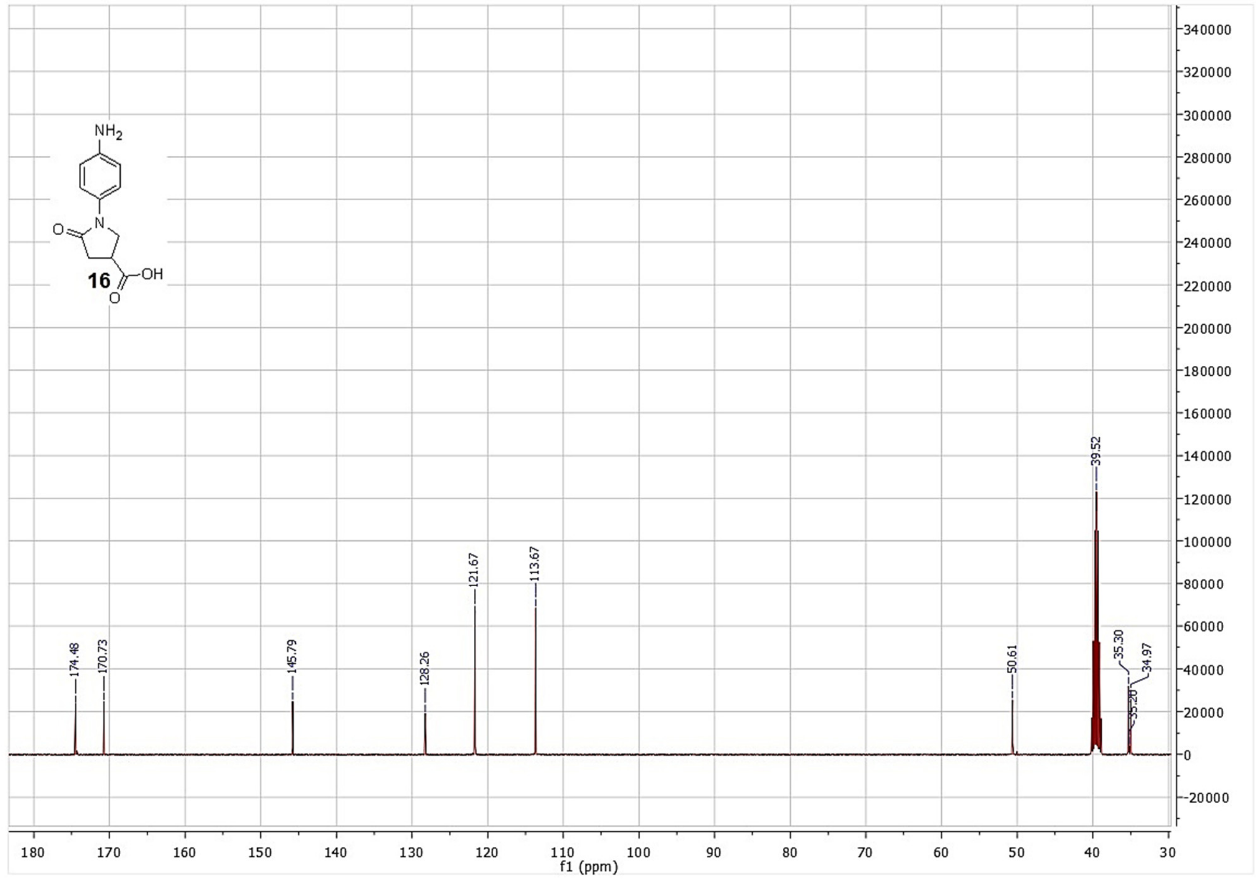

Figure S20. ^{13}C NMR of compound **16.**

1-(4-Aminophenyl)-5-oxopyrrolidine-3-carbohydrazide **(17)**.

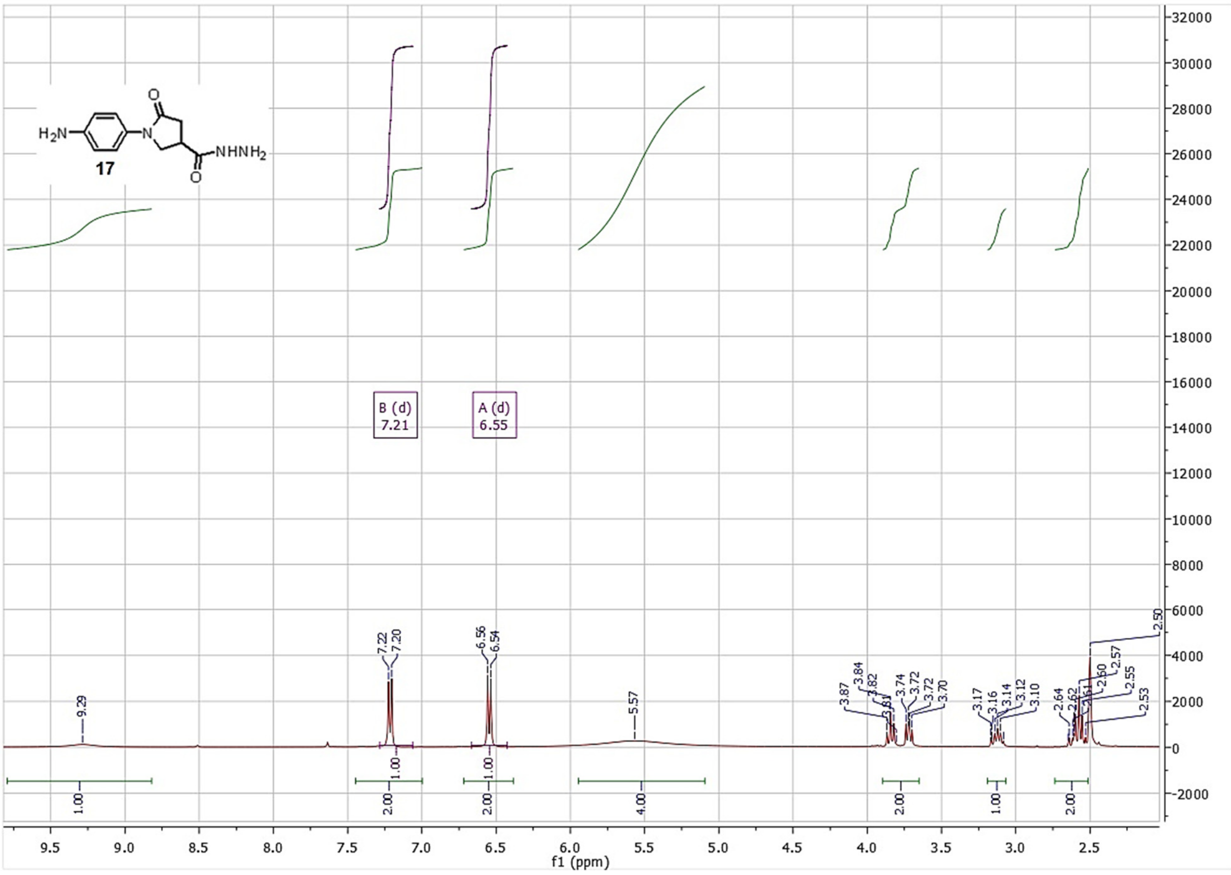

Figure S21. ^{1}H NMR of compound **17.**

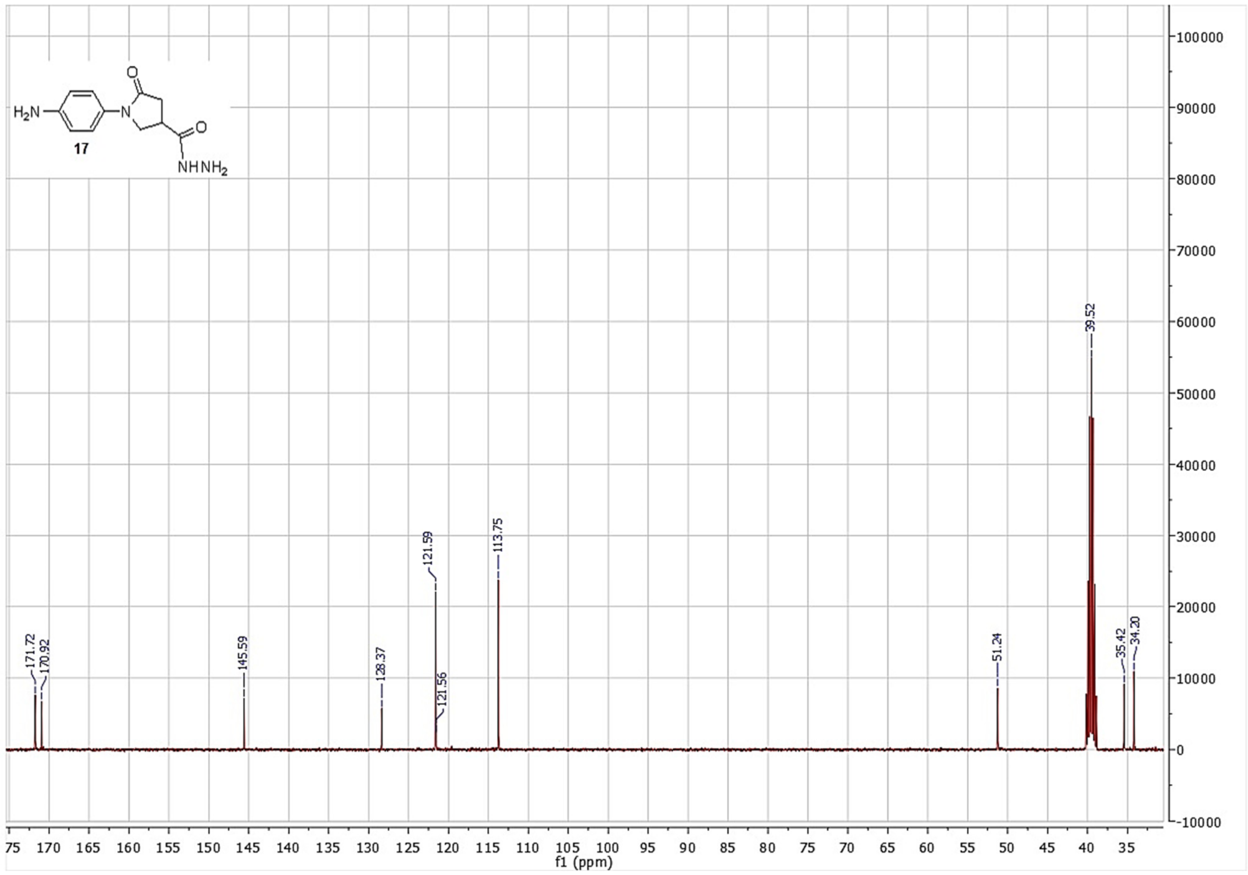

Figure S22. ^{13}C NMR of compound **17.**

1-(4-Aminophenyl)-4-(1H-benzo[d]imidazol-2-yl)pyrrolidin-2-one **(18)**.

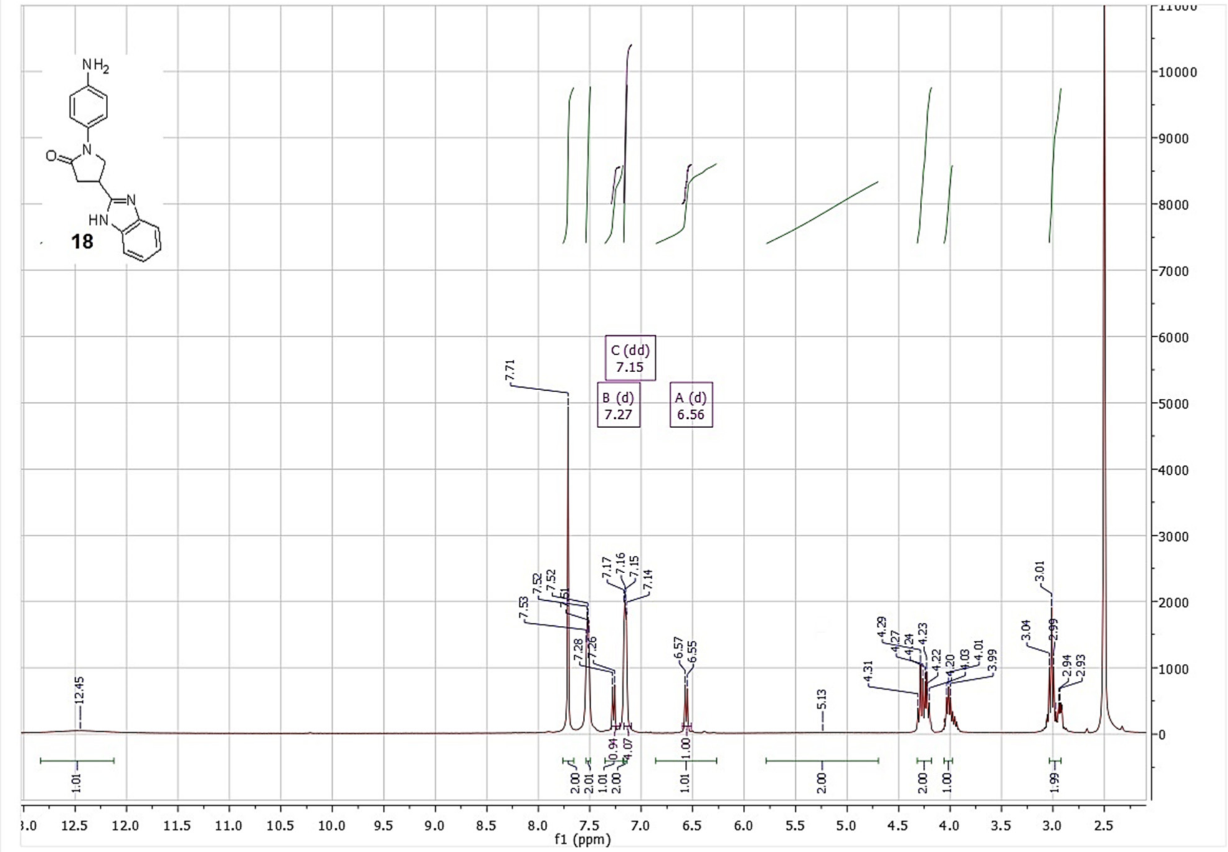

Figure S23. ^{1}H NMR of compound **18.**

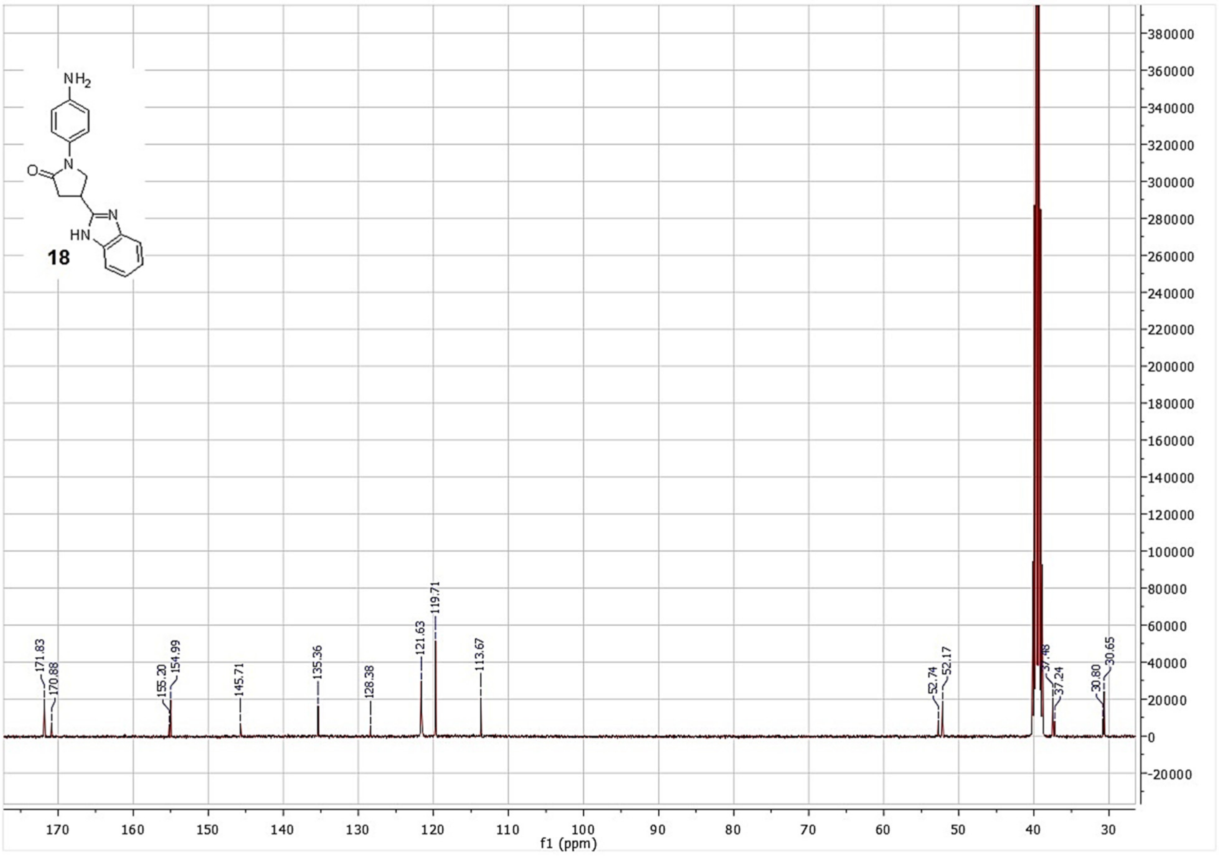

Figure S24. ^{13}C NMR of compound **18.**

1-(4-(2,5-Dimethyl-1H-pyrrol-1-yl)phenyl)-5-oxpyrrolidine-3-carboxylic acid **(19)**.

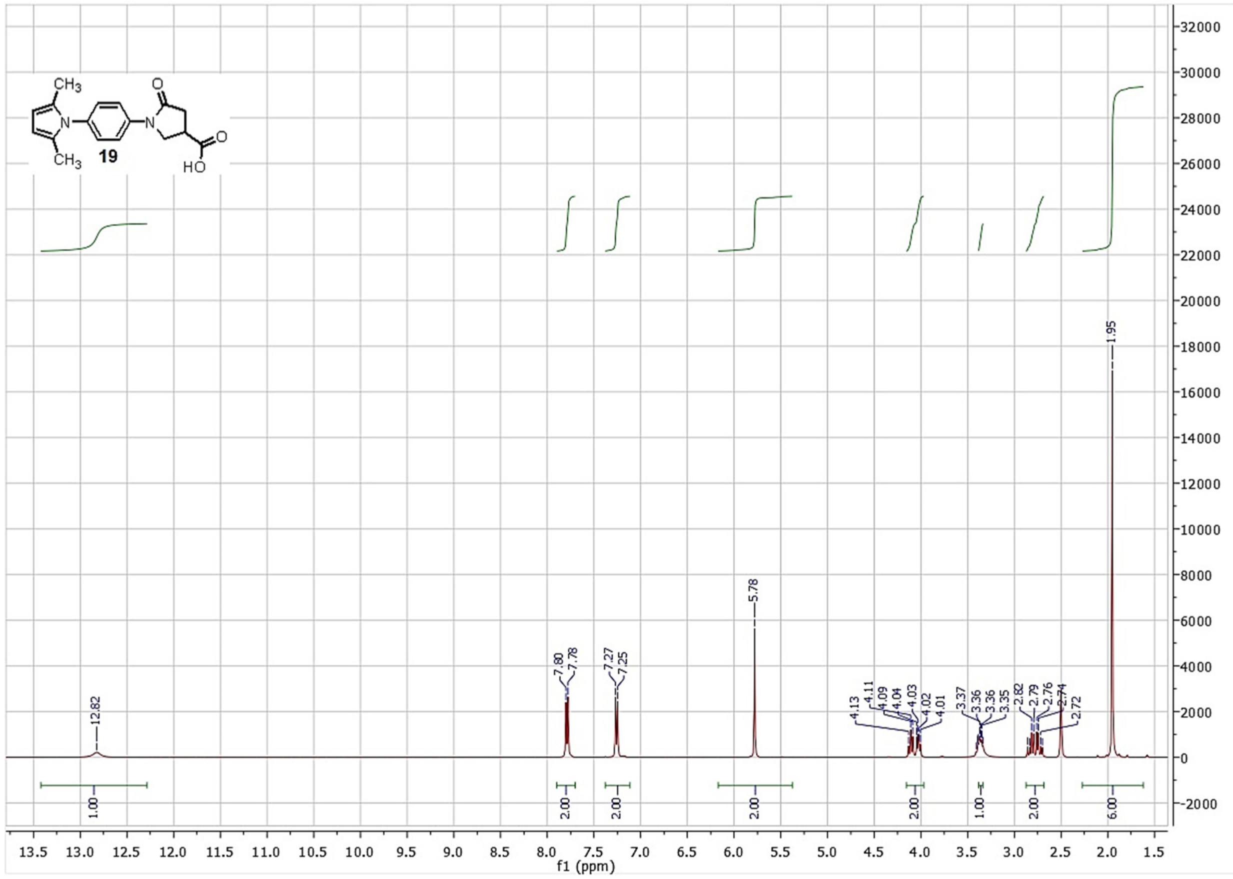

Figure S25. ^{1}H NMR of compound **19.**

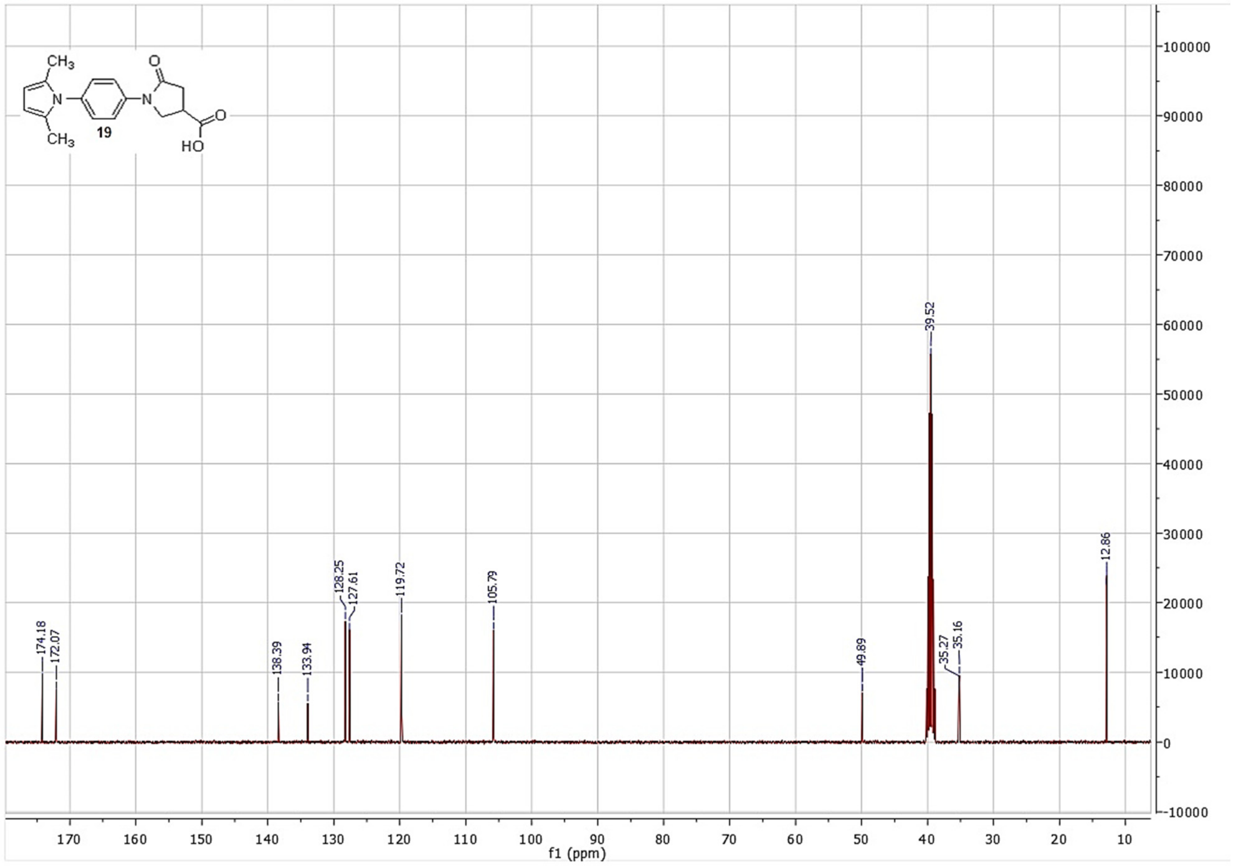

Figure S26. ^{13}C NMR of compound **19.**

5-Oxo-N′-(thiophen-2-ylmethylene)-1-(4-((thiophen-2-ylmethylene)amino)phenyl)pyrrolidine-3-carbohydrazide **(20)**.

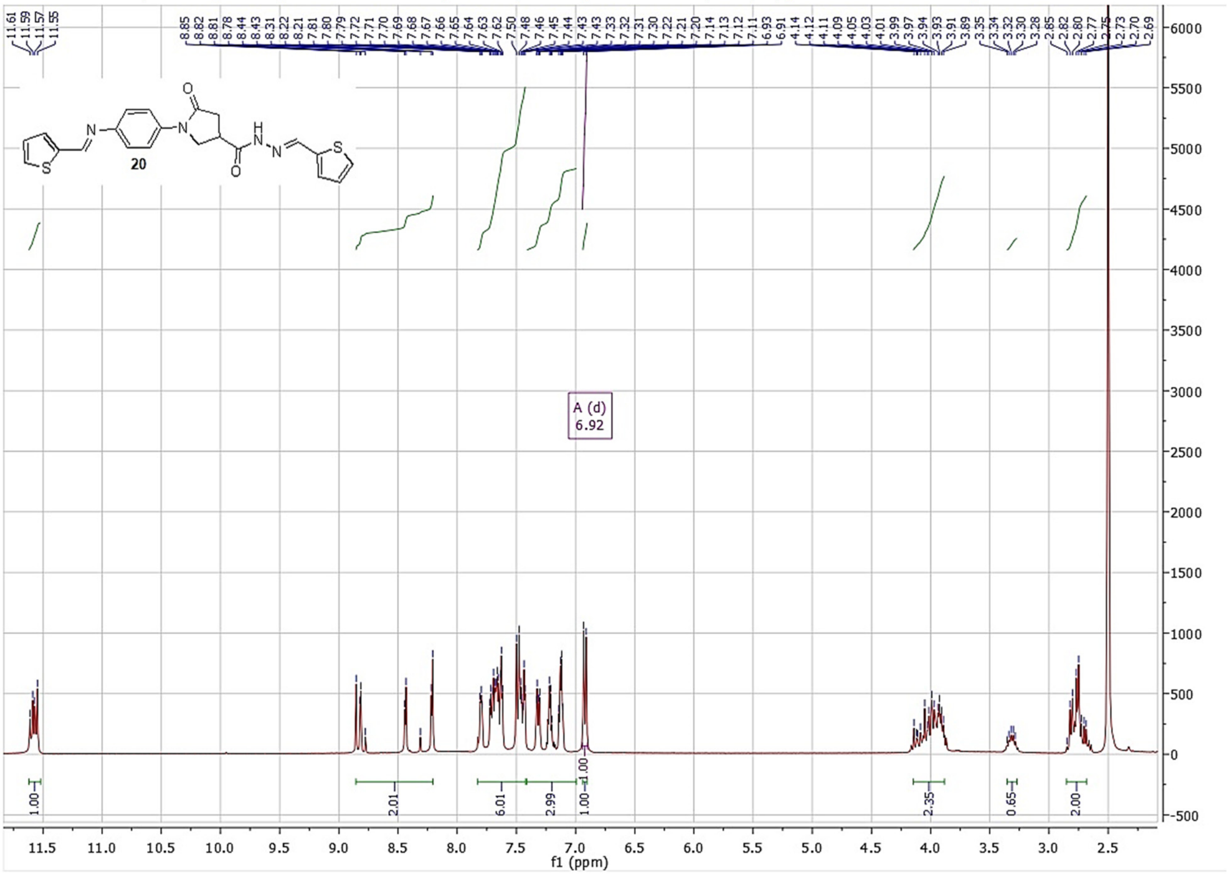

Figure S27. ^{1}H NMR of compound **20.**

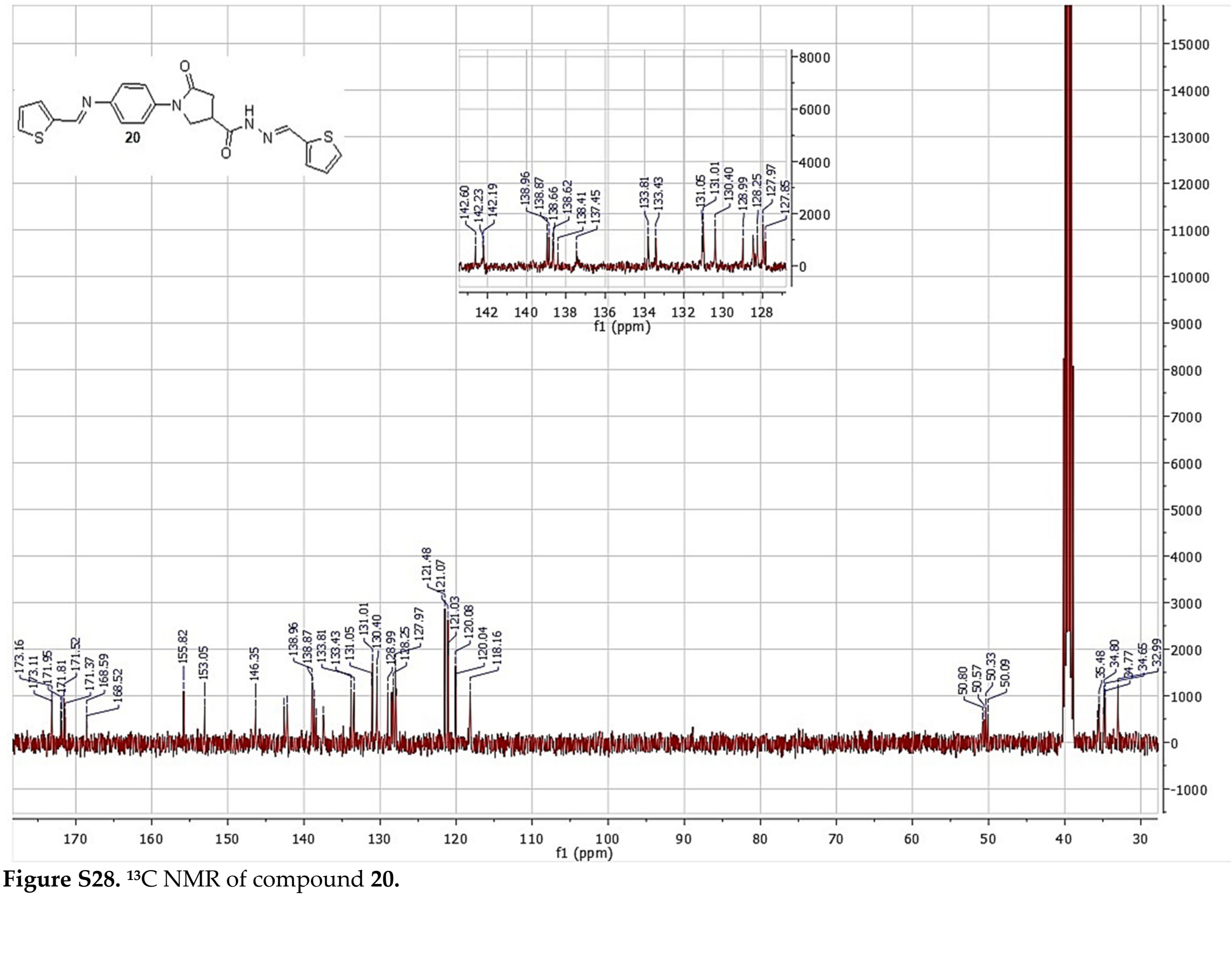

Figure S28. ^{13}C NMR of compound **20.**

N′-((5-nitrothiophen-2-yl)methylene)-1-(4-(((5-nitrothiophen-2-yl)methylene)amino)phenyl)-5-oxopyrrolidine-3-carbohydrazide **(21)**.

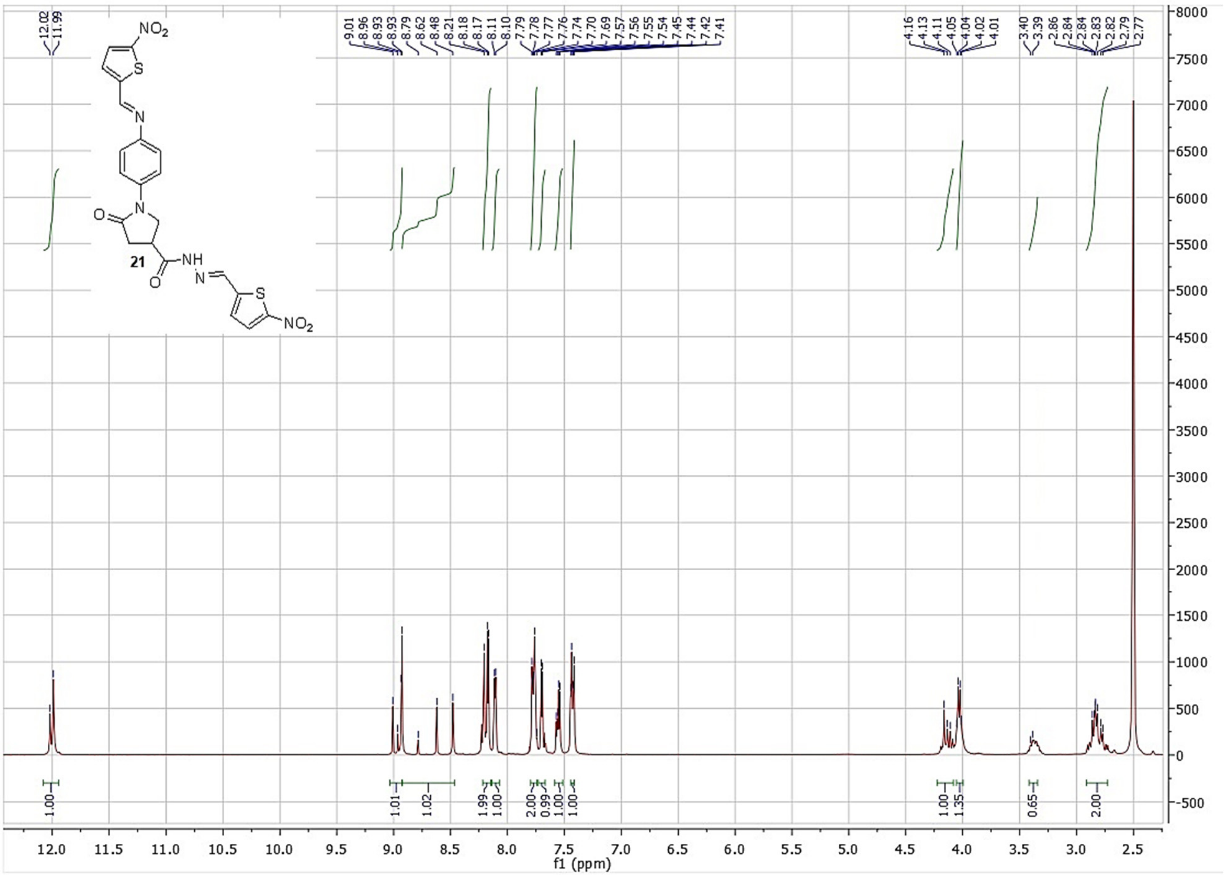

Figure S29. ^{1}H NMR of compound **21.**

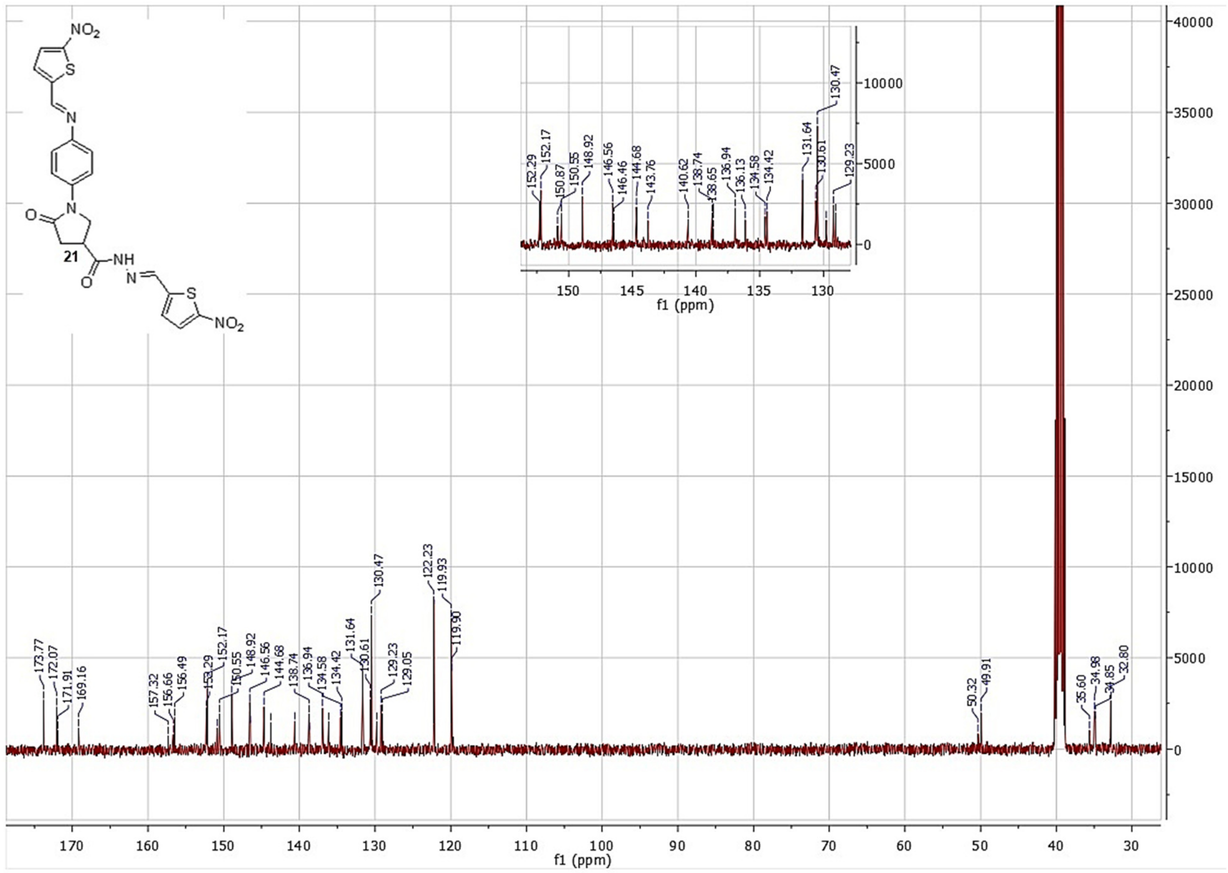

Figure S30. ^{13}C NMR of compound **21.**

N-(2,5-dimethyl-1H-pyrrol-1-yl)-1-(4-(2,5-dimethyl-1H-pyrrol-1-yl)phenyl)-5-oxopyrrolidine-3-carboxamide **(22)**.

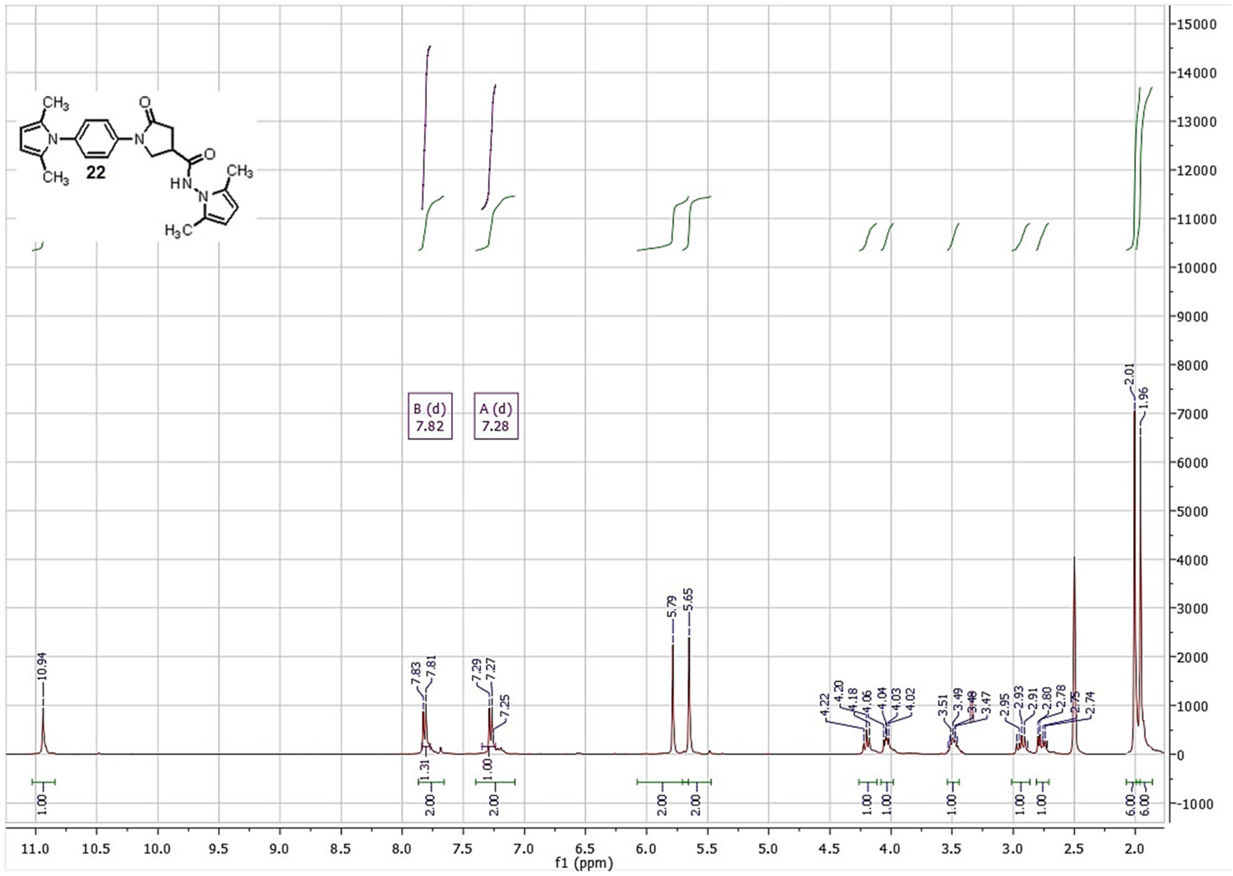

Figure S31. ^{1}H NMR of compound **22.**

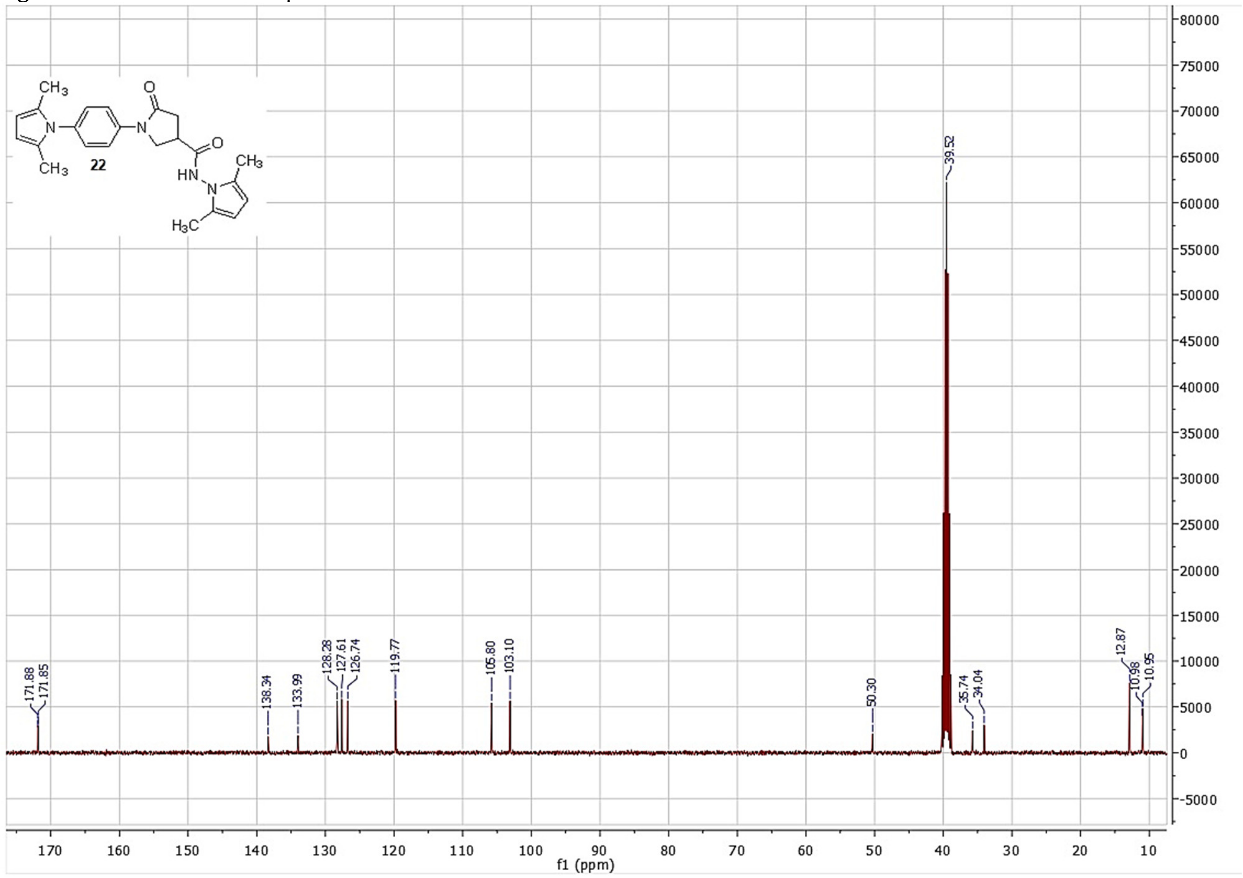

Figure S32. ^{13}C NMR of compound **22.**

Table S1. The minimal inhibitory concentration of compounds **2, 4–22** against tested bacterial pathogens.

	**MIC (μg/mL)**					
**Compound**	***S. aureus MRSA***	***K. pneumoniae KPC-1***	***K. pneumoniae NDM-1***	***A. baumannii NDM-1***	***P. aeruginosa OprD* porin loss**	***E. coli Mcr-1***
**2**	>64	>64	>64	>64	>64	>64
**4**	>64	>64	>64	>64	>64	>64
**5**	>64	>64	>64	>64	>64	>64
**6**	>64	>64	>64	>64	>64	>64
**7**	>64	>64	>64	>64	>64	>64
**8**	>64	>64	>64	>64	>64	>64
**9**	>64	>64	>64	>64	>64	>64
**10**	>64	>64	>64	>64	>64	>64
**11**	>64	>64	>64	>64	>64	>64
**12**	>64	>64	>64	>64	>64	>64
**13**	>64	>64	>64	>64	>64	>64
**14**	>64	>64	>64	>64	>64	>64
**15**	>64	>64	>64	>64	>64	>64
**16**	>64	>64	>64	>64	>64	>64
**17**	>64	>64	>64	>64	>64	>64
**18**	>64	>64	>64	>64	>64	>64
**19**	>64	>64	>64	>64	>64	>64
**20**	>64	>64	>64	>64	>64	>64
**21**	1	>64	>64	>64	>64	>64
**22**	>64	>64	>64	>64	>64	>64
**Vancomycin**	2	N/A	N/A	N/A	N/A	N/A
**Meropenem**	N/A	8	16	8	4	1
**Colistin**	N/A	1	4	16	4	32

Abbreviations: N/A – not applicable; MRSA – methicillin resistant *S. aureus*; KPC- *Klebsiella pneumoniae* carbapenemase 1; NDM-1 – New Delhi metallo beta lactamase 1; Mcr-1 – mobile colistin resistance.
